# Simple lysis of bacterial cells for DNA-based diagnostics using hydrophilic ionic liquids

**DOI:** 10.1038/s41598-019-50246-5

**Published:** 2019-09-30

**Authors:** Roland Martzy, Katharina Bica-Schröder, Ádám Márk Pálvölgyi, Claudia Kolm, Stefan Jakwerth, Alexander K. T. Kirschner, Regina Sommer, Rudolf Krska, Robert L. Mach, Andreas H. Farnleitner, Georg H. Reischer

**Affiliations:** 1 TU Wien, Institute of Chemical, Environmental & Bioscience Engineering, Molecular Diagnostics Group, Department of Agrobiotechnology (IFA-Tulln), Tulln, Austria; 2 ICC Interuniversity Cooperation Centre Water & Health, Vienna, Austria; 3 TU Wien, Institute of Applied Synthetic Chemistry, Research Group for Sustainable Organic Synthesis and Catalysis, Vienna, Austria; 4 Medical University Vienna, Institute for Hygiene and Applied Immunology, Unit Water Hygiene, Vienna, Austria; 5 University of Natural Resources and Life Sciences Vienna (BOKU), Department of Agrobiotechnology (IFA-Tulln), Tulln, Austria; 6 Institute for Global Food Security, School of Biological Sciences, Queen’s University Belfast, Northern Ireland, United Kingdom; 7 TU Wien, Institute of Chemical, Environmental & Bioscience Engineering, Research Area Biochemical Technology 166/5, Vienna, Austria; 8 Karl Landsteiner University of Health Sciences, Department for Pharmacology, Physiology and Microbiology, Research Area Water Quality and Health, Krems, Austria; 9 TU Wien, Institute of Chemical, Environmental & Bioscience Engineering, Research Area Biochemical Technology, Research Group of Environmental Microbiology and Molecular Diagnostics, Vienna, Austria

**Keywords:** Applied microbiology, Bacteriology, Clinical microbiology, Water microbiology

## Abstract

The extraction of nucleic acids from microorganisms for subsequent molecular diagnostic applications is still a tedious and time-consuming procedure. We developed a method for the rapid preparation of genomic DNA from bacteria based on hydrophilic ionic liquids (ILs). First, we tested eight ILs in different buffer systems for their inhibitory effects on quantitative PCR. The cell lysis potential of different IL/buffer combinations was assessed by application on *Enterococcus faecalis* as a model organism for Gram-positive bacteria. The two best ILs, choline hexanoate and 1-ethyl-3-methylimidazolium acetate, were compared with the reference enzymatic method and two commercial DNA extraction kits. All methods were evaluated on four Gram-positive and four Gram-negative bacterial species that are highly relevant for environmental, food, or clinical diagnostics. In comparison to the reference method, extraction yields of the IL-based procedure were within one order of magnitude for most of the strains. The final protocol for DNA extraction using the two ILs is very low-cost, avoids the use of hazardous chemicals and can be performed in five minutes on a simple heating block. This makes the method ideal for high sample throughput and offers the opportunity for DNA extraction from bacteria in resource-limited settings or even in the field.

## Introduction

The field of microbial molecular diagnostics comprises various methods for the specific detection of nucleic acids (NAs) from different microorganisms^1,2^ – e.g., human pathogens in clinical and environmental samples^3–5^, faecal indicator bacteria in water^6,7^, or harmful microbial agents in food and feed^8,9^. However, to detect the desired sequence of a certain NA (DNA or RNA), preceding steps are necessary to isolate the genetic material from the respective cells. These steps typically involve the lysis of the cells, the purification of the NAs to remove other cell components, inhibitory substances or degrading enzymes, and the subsequent recovery of the desired NAs. Common methods for cell lysis involve thermal, chemical, enzymatic, or mechanical treatment of the cells or a combination of those^1^. The purification of the NAs is, in most cases, achieved either by precipitation followed by washing steps, or by column-based purification protocols. State-of-the-art extraction procedures for microorganisms traditionally use incubation steps with enzymes such as lysozyme and proteinase K to digest cell wall components and interfering proteins, respectively. Hazardous chemicals such as phenol and chloroform^10^, or commercial kits are then used for NA purification, depending on the area of application and the matrix in which the cells are investigated. These methods are well established and result in high quality DNA or RNA, but they are often very laborious, time-consuming and cost-intensive, or suffer from insufficient and inconsistent yields of NAs. These disadvantages are even more significant when considering applications of molecular diagnostics in low-resource settings (e.g., developing countries), since such sample preparation procedures strongly hinder the implementation of molecular diagnostics in many regions of the world. Hence, a more efficient and user-friendly DNA extraction method could also promote the progress of molecular point-of-care detection methods, which are still dependent on sophisticated laboratory infrastructure. In recent years, novel approaches for the extraction of DNA from biological samples have been using ionic liquids (ILs), which are organic salts that are liquid at temperatures below 100 °C or even at room temperature. Their properties allow for the dissolution of a variety of biopolymers such as (ligno-)cellulose or chitin, which is already exploited in the chemical industry^11^. In addition, it has been shown that ILs are able to efficiently lyse different types of cells or separate them from various biological materials. In this way, the DNA from organisms such as maize, meat, viruses, or Gram-negative bacteria can be extracted within minutes^12–15^. However, the cell wall of Gram-positive bacteria is protected by a hardy peptidoglycan layer^14^, which remained unaffected by the treatment with hydrophobic ILs and high temperatures^14^. For this purpose, enzymes such as proteinase K are still required to break up these cell walls, making extraction protocols tedious and time consuming. Therefore, a rapid extraction method that consistently generates high DNA yields and that can be applied to both Gram-positive and Gram-negative bacteria without additional enzymatic treatment would be of great benefit to save time, money and allow higher sample throughput.

The aim of this study was the development of a novel IL-based method for the rapid lysis of Gram-positive bacteria that can be carried out with minimal laboratory equipment for subsequent DNA-based diagnostics. To this end, we selected *Enterococcus faecalis* as a model organism and tested a selection of hydrophilic ionic liquids on their cell lysis potential. After optimizing the reaction conditions regarding buffer system, IL concentration, temperature and incubation time, the novel DNA preparation method was compared to state-of-the-art protocols and commercial DNA extraction kits with different bacterial targets.

## Results and Discussion

### Selection of candidate ionic liquids

A set of eight ionic liquids with a choline or 1,3-dialkylimidazolium core structure and variable anions was chosen for this study (Table 1). The selection is based on our previous experience in the direct extraction of DNA, as choline-based ionic liquids were particularly suitable for the extraction of DNA from meat samples^13^. Moreover, choline-based ionic liquids with carboxylate cations are typically considered as environmentally benign and bio-derived ionic liquids with low toxicity^16,17^. As our previous studies showed a strong influence of the anion on the extraction performance, but also on DNA amplification in the qPCR reaction, we included three carboxylate anions with variable chain lengths as well as a phosphate-based anion in the selection. This pool was complemented by four imidazolium-based ionic liquids with either halide, acetate or phosphate anions due to their outstanding and well-known ability for the dissolution of biopolymers such as cellulose^18^.

**Table 1:** Ionic liquids and their abbreviations as used in this study.

Compound	Abbreviation	Structure
1-Ethyl-3-methylimidazolium acetate	[C_2_mim]OAc	
1-Ethyl-3-methylimidazolium dimethylphosphate	[C_2_mim]Me_2_PO_4_	
1-Ethyl-3-methylimidazolium chloride	[C_2_mim]Cl	
1-Hexyl-3-methylimidazolium chloride	[C_6_mim]Cl	
Choline formate	[Cho]Fmt	
Choline lactate	[Cho]Lac	
Choline hexanoate	[Cho]Hex	
Choline dibutylphosphate	[Cho]DBP	

### Influence of ionic liquids and buffer systems on qPCR

**Figure 1:**
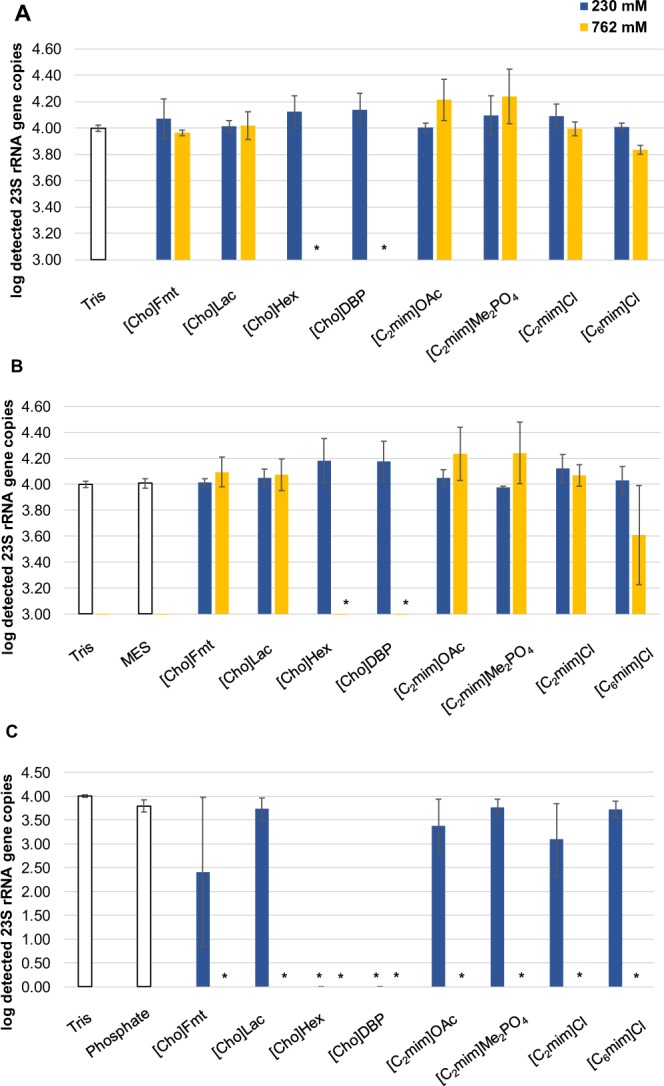
Results of the qPCR analysis of eight ionic liquids in different concentrations spiked with a DNA plasmid standard (10^4^ DNA target copies in each reaction); (*) not detected. The ILs were solved in. (**A**) Tris buffer. (**B**) MES buffer, and (**C**) sodium phosphate buffer. The whiskers indicate the standard deviations of the qPCR triplicates.

In a first step, we investigated the inhibitory effects of the selected ILs on the successive molecular diagnostic methods. We decided to use quantitative PCR because it allows easy observation of reductions in amplification efficiency. Strong inhibition of the amplification reaction would make the respective ILs unsuitable as our DNA preparation method. We determined the tolerable concentration of the ILs by adding them to the qPCR reactions in different concentrations. We tested three different buffer systems that were previously reported for similar applications to dissolve and dilute the ILs, namely tris(hydroxymethyl)aminomethane (Tris, 10 mM, pH 8.0); 2-(N-morpholino)ethanesulfonic acid (MES, 50 mM, pH 6.0); and sodium phosphate (50 mM, pH 8.5)^13^. Subsequently, we spiked qPCR reactions containing 10^4^ copies of *Enterococcus* spp. 23S rRNA gene fragment on DNA plasmid with four different concentrations of the ILs (230 mM; 762 mM; 1250 mM; 2300 mM). The 23S rRNA gene copies were then determined by the *Enterococcus*-specific qPCR assay Method 1611 of the USEPA (ENT-qPCR; Fig. 1)^19^. No qPCR inhibition was observed with 230 mM final concentration of ILs solved in the Tris and MES buffers. At concentrations of 762 mM, [Cho]Hex and [Cho]DBP already completely inhibited the amplification, and [C_6_mim]Cl substantially interfered with the reaction. In contrast, the sodium phosphate buffer system interfered with the qPCR reactions already without ILs and partially or completely inhibited the amplification reaction at IL concentrations of 230 mM and 762 mM (Fig. 1C). Ionic liquid concentrations of 1250 mM and 2300 mM completely inhibited the amplification in all three buffer systems. Based on these results, we excluded the phosphate buffer system from further experiments and continued the method development with the ionic liquids diluted in Tris and MES buffers, respectively.

### Cell lysis experiments

In a next step, we tested the effect of the ILs on the lysis of Gram-positive bacterial cells. For this purpose, we used *Enterococcus faecalis* type strain NCTC 775 as model organism for Gram-positive bacteria. *Enterococcus* species are very commonly occurring in nature and hold important relevance in clinical, food, and environmental diagnostics. Furthermore, the ENT-qPCR assay represents a reliable method that is recommended by the U.S. EPA for the routine monitoring of bathing water quality in marine systems. First, we cultivated the *Enterococcus* cells in liquid media and counted the cells using fluorescence microscopy at different time points to determine the optical cell density at 670 nm (OD_670_) and the corresponding cell number (data not shown). For the cell lysis experiments, we harvested the cells after five hours at OD_670_ = 0.2, corresponding to approximately 10^8^ cells per ml as determined by fluorescence microscopy. This approach ensured a reproducible condition of an early growth phase where most of the cells were dividing and the percentage of dead cells was at a low level^20^.

**Figure 2:**
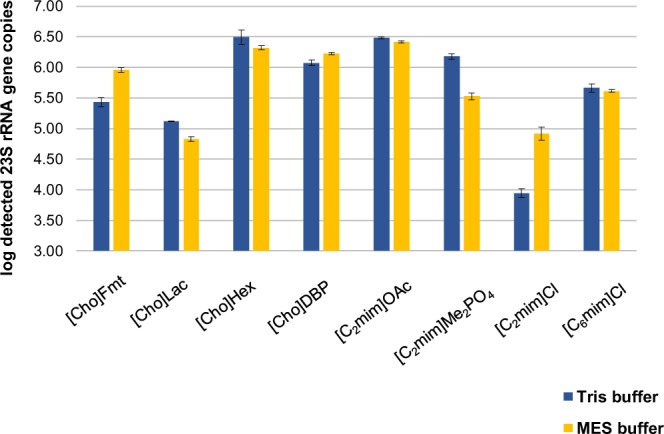
*Enterococcus* 23S rRNA gene copies (log_10_-transformed) measured by qPCR in triplicates after cell lysis experiments with eight different ILs (90% w/v) diluted with Tris or MES buffer.

To remove free DNA as well as nutrient medium from the liquid culture, the cells were pelleted, washed, and resuspended in the same buffer system that we subsequently used for the cell lysis experiments (Tris and MES). For a first screening, we incubated the resuspended cells with each IL at a concentration of 90% w/v for 30 minutes at 95 °C. To alleviate PCR inhibition caused by high IL concentrations, we diluted the crude extracts with the corresponding buffer in a 1:20 ratio and applied the ENT-qPCR assay to quantify the released DNA target molecules (Fig. 2). We detected approximately log 6.49 ± 0.11 and log 6.48 ± 0.02 23 S rRNA gene copies in 2.5 µl of the DNA extracts that resulted from the two best performing ionic liquids, [Cho]Hex and [C_2_mim]OAc. The DNA yields obtained with these ILs in MES buffer were slightly lower, which is why we decided to continue the method development only with [Cho]Hex and [C_2_mim]OAc in the Tris buffer system.

**Figure 3:**
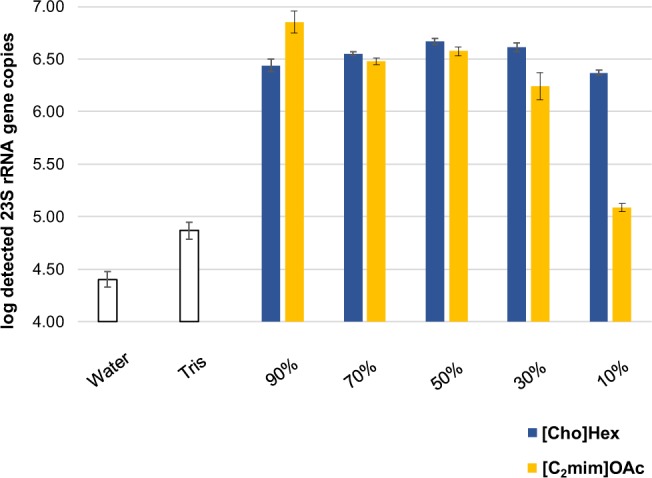
*Enterococcus* 23S rRNA gene copies(log_10_-transformed) measured by qPCR after cell lysis experiments with varying concentrations of [C_2_mim]OAc and [Cho]Hex. The cell lysis experiments were carried out in five replicates for each condition.

To investigate the influence of varying IL concentrations on the cell lysis efficiency, we applied the selected ILs at concentrations ranging from 90% to 10% to the same cell lysis procedure as described above. As a reference, we tested the lysis efficiency of the same cell suspension in pure double-distilled water and Tris buffer, respectively (Fig. 3). The efficiency of [C_2_mim]OAc almost steadily decreased with its respective concentration, while the performance of [Cho]Hex slightly improved towards a concentration of 50%, only decreasing with lower concentrations of 30% and 10%. As expected, heating the cell suspension with double-distilled water or Tris buffer resulted in a much lower yield of extracted DNA. Due to the high yields obtained with the respective IL concentrations, we selected 90% [C_2_mim]OAc and 50% [Cho]Hex as the candidates for all subsequent experiments.

Finally, we replicated the cell lysis procedures ten times for both ionic liquids to learn about the variability of the respective DNA yields. The results were compared to a ten-fold replication of the cell lysis with double-distilled water (data not shown). The cell lysis with 90% [C_2_mim]OAc yielded log 6.69 ± 0.13 target DNA molecules in a measured volume of 2.5 µl crude extract, whereas 50% [Cho]Hex yielded log 6.67 ± 0.08 target DNA molecules in the same volume.

### Optimization of the cell lysis conditions for the selected ILs

**Figure 4:**
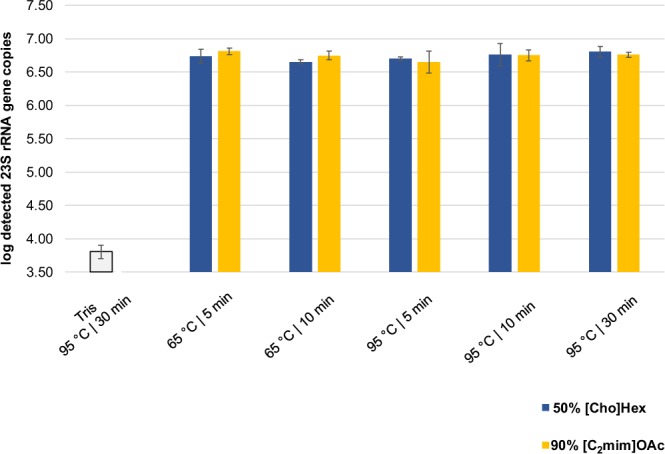
*Enterococcus* 23S rRNA gene copies (log_10_-transformed) measured by qPCR after a five-fold replication of the cell lysis experiments submitted to varying temperatures and incubation periods.

To further optimize the cell lysis procedure, we incubated the *E*. *faecalis* cells with the selected ILs at 95 °C and 65 °C for three different incubation times in five replicates each. Figure 4 shows that no differences between the variations occurred, therefore an incubation time and temperature of 5 min at 65 °C was selected.

### Comparison of the selected ionic liquids with three conventional methods based on eight bacterial reference strains

Finally, we assessed the performance of the IL-based DNA preparation methods in comparison with three established procedures. To represent Gram-positive bacteria, we selected *Clostridium perfringens*, *Bacillus subtilis*, and *Staphylococcus aureus* in addition to *Enterococcus faecalis*. Furthermore, we also tested the ILs on the Gram-negative species *Escherichia coli*, *Legionella pneumophila*, *Pseudomonas aeruginosa*, and *Vibrio cholerae* as model organisms. Some of these strains are commonly used as indicator bacteria, whereas others represent widespread human pathogens that are clinically relevant or are attributed to food spoilage, respectively. In addition to the established IL-based method, we extracted the selected strains with the QIAamp DNA Mini Kit from Qiagen and the Wizard Genomic DNA Purification Kit from Promega, as well as with an enzymatic method with subsequent DNA purification using phenol and chloroform (reference method)^21^. All three methods for cell lysis are based on the incubation with lysozyme and proteinase K, followed either by precipitation or column-based purification of the isolated DNA. Including hands-on time, these steps took up to three hours per sample, depending on the method and the nature of the cells (compare Table 2).

**Table 2:** Overview on approximate prices and durations per sample, calculated for the five extraction methods that were used in this study.

Extraction method	Price per sample	Duration per sample
[Cho]Hex	0.73€^1^	5 min (Gram^+^ and^−^)
[C_2_mim]OAc	1.14€^2^	5 min (Gram^+^ and^−^)
Phe/Chl	1.46€	180 min (Gram^+^) 120 min (Gram^−^)
Promega	2.31€^3^	134–214 min (Gram^+^) 102–152 min (Gram^−^)
Qiagen	3.10€^4^	87 min (Gram^+^) 22 min (Gram^−^)

The prices also include the costs for pipette tips and reaction tubes but neglect the personnel costs that arise from the working hours. The durations reflect the sum of all incubation and centrifugation steps, but do not include buffer preparation and general handling, such as pipetting, centrifuge (un)loading, or reaction tube labelling. Washing of the cells was part of the sample preparation and was the same for all extraction methods, which is why this was not considered in the time calculations.

^1^Custom synthesis by Iolitec (Heilbronn, Germany), based on the minimum amount of 50 g (offer from February 6, 2018); ^2^689483 from Sigma-Aldrich/Merck, based on the 50 g packaging size (calculated with the price from September 14, 2018); ^3^Article A1125 from Promega (September 14, 2018); ^4^Article 51306 from Qiagen (September 14, 2018).

**Figure 5:**
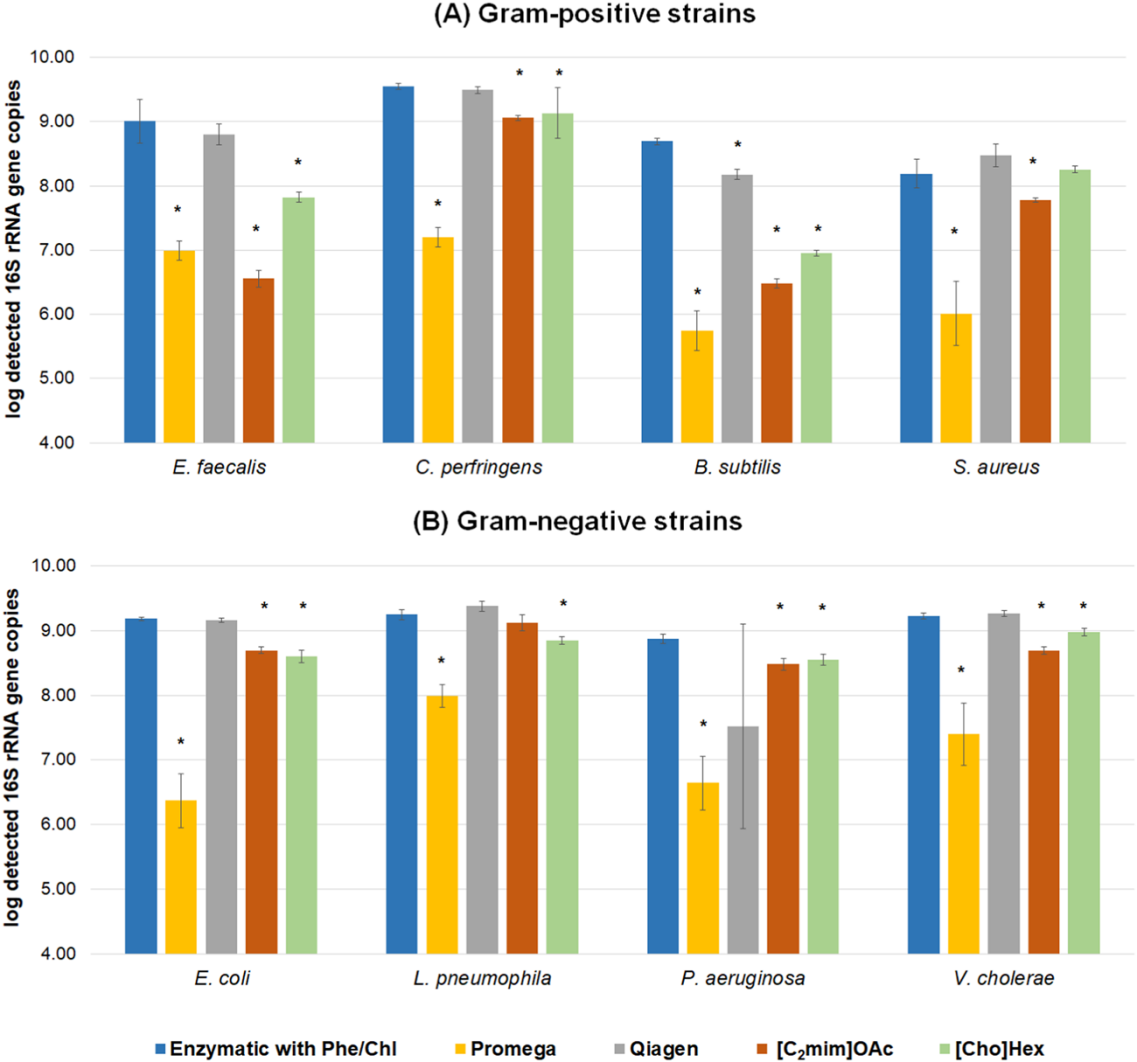
16S rRNA gene copies in the DNA extracts obtained from five extraction methods applied to (**A**) four Gram-positive, and (**B**) four Gram-negative bacterial reference strains. All strains were extracted three times with each individual method. The subsequent qPCR reactions were carried out in duplicate for each extraction, resulting in six independent measurements per strain and method. (*) The asterisks indicate a statistically significant difference compared to the phenol/chloroform extraction, which we defined as the reference method.

Following DNA isolation, the samples were analysed by qPCR to quantify the number of extracted cells and compare the methods to each other (Fig. 5). To avoid the use of eight different species-specific qPCR assays while ensuring the comparability of the results, we analysed all DNA extracts with a qPCR assay targeting a 16S rRNA gene fragment that is universal to all bacteria (denoted as 16S-qPCR in this study). Although the used bacteria differ in their 16S rRNA operon copy number, the results obtained from the employed methods can be compared in a relative manner for each individual species.

To test for inhibitory effects, we additionally analysed 1:4 dilutions of all extracts and plotted the results for each individual method against the results from the corresponding undiluted extracts. In case of the ILs, the 1:20 dilutions were further diluted to obtain a final 1:80 dilution. No inhibitory effects were evident in the undiluted extracts obtained with the Phe/Chl extraction and the commercial kits. We observed no difference in the 1:20 and 1:80 dilutions for most IL extracts, whereas some samples yielded slightly better results with the higher dilution, indicating weak inhibition. Whenever this was the case, we prepared 1:320 dilutions of the respective samples and used the results from the 1:80 and 1:320 dilutions as basis for the calculations as they were no longer indicating inhibitory effects. All qPCR runs showed an efficiency between 90 and 105% (see Supplementary Figs S1 and S2 for exemplary standard curves).

For the evaluation of the results, we set the enzymatic extraction as our reference to which the other methods were compared in a relative manner. As can be seen from the results, the enzymatic extraction was in many cases superior to the other methods for all Gram-positive strains tested. However, except for the extraction of *B*. *subtilis*, the Qiagen extraction kit performed equally well, with no statistically significant difference in the results as compared to the phenol/chloroform extraction. Unexpectedly, the Promega kit yielded approximately two orders of magnitude lower DNA concentration than the enzymatic extraction for all eight strains. Regarding the ionic liquids, [Cho]Hex was superior to [C_2_mim]OAc in the lysis of Gram-positive strains, whereas the results were balanced in view of the Gram-negative strains. However, as for *L*. *pneumophila*, [C_2_mim]OAc outperformed [Cho]Hex, showing no statistically significant differences in the results as compared to Phe/Chl. In comparison to the reference method, choline hexanoate showed a slightly better performance for *S*. *aureus* and similar results for *C*. *perfringens* as well as all Gram-negative strains within one order of magnitude. Both *E*. *faecalis* and *B*. *subtilis* could not be lysed as efficiently by the ionic liquids as with the enzymatic extraction method. Nevertheless, [Cho]Hex outcompeted the Promega extraction kit regarding all strains tested.

To see if the ionic liquids have an effect on the specificity of the a qPCR assay, we spiked DNA samples from the 8 strains obtained from the phenol/chloroform extraction with ILs at the concentrations present in the IL extracts and subjected 1:20 dilutions of these samples to ENT-qPCR analysis (data not shown). As for the *E*. *faecalis* extracts, we detected a number of gene copies that is comparable to the one measured with the 16S qPCR assay. The extracts of some of the other strains also yielded sporadic positive signals, but always below the assay limit of detection, i.e., less than seven copies per reaction and therefore 3–4 orders of magnitude lower than the general 16S rRNA gene copy numbers. At this point, it should additionally be noted that the USEPA 23S qPCR assay itself is not 100% specific, as previously demonstrated by the authors^6^.

Taken all together, the preparation of genomic DNA from bacteria after cell lysis with ionic liquids has several advantages over common enzymatic methods or commercial kits. First, the developed method solely depends on a single incubation step of five minutes, compared to the numerous incubation and centrifugation steps which amount to two to three hours for the extraction with most traditional methods. Consequently, the handling of the procedure using ILs is much simpler in view of parallel extraction runs, thus allowing a high sample throughput. Furthermore, there is no need for a fume hood or even centrifuges, but the reaction can be carried out on a simple heating block or in a water bath. To additionally provide an economic perspective, we calculated the prices for the extraction of a single sample with each individual method (Table 2). Including consumables such as pipette tips and reaction tubes, the preparations with [Cho]Hex or [C_2_mim]OAc are cheapest at an approximate price of 0.73€ or 1.14€ per reaction, respectively. In this regard, it should be noted that the given prices for ionic liquids were taken from small sample sizes for laboratory scale applications; it can be expected that prices will be considerably lower when the ionic liquids are produced on a larger scale^22^. In comparison, the commercial kit from Qiagen is the most expensive of the five methods used, with a price of 3.10€ for the extraction of a single sample.

In times of massive environmental pollution from toxins and plastic waste, one must also consider the use of volatile organic solvents in traditional enzymatic methods, as well as the excessive packaging of consumables that comes with some commercial kits. In contrast, the DNA preparation with ionic liquids is carried out in a single tube, hence involving fewer pipetting steps and less material, thereby offering a low environmental footprint, especially in combination with the use of biodegradable molecules such as choline hexanoate.

While several studies addressed the long-term stability of ionic liquids under thermal or thermal-oxidative conditions, relatively little information is available on the shelf life stability upon storage. However, since most ionic liquids do not exhibit a significant mass loss at temperatures up to 200 °C^23^, we do not anticipate any problem, particularly when stored under inert atmosphere. This is also in accordance with our investigations towards the stability of one of the ionic liquids selected in here (choline hexanoate) that was stored for 16 months at room temperature on a bench shelf. Based on the results from NMR spectroscopy (Supplementary Figs S12 and S13), no significant change in the purity of the ionic liquid was observed, indicating excellent stability during storage at ambient conditions.

A major disadvantage of the IL-based DNA preparation arises from the dilution step necessary for subsequent downstream applications, which indirectly influences the detection limit of analytical methods such as qPCR (see Supplementary Material for an exemplary calculation). Although this might not make a significant impact on the analysis of bacteria that are cultivated prior to DNA extraction, it becomes relevant when samples are analysed that contain a multitude of different microorganisms. In this case, there are many bacterial species that cannot be enriched by cultivation, which might result in false-negative signals if the targeted DNA is diluted below the detection limit of the respective analysis method. However, to overcome this drawback, it might be feasible to employ DNA-binding columns or silica coated magnetic beads to purify the nucleic acids from the crude extract. Moreover, the group of Anderson demonstrated the rapid extraction of DNA from aqueous solutions by applying magnetic ionic liquids^24^, for which they proposed sample preparation techniques for the subsequent downstream analysis with PCR^25,26^.

In conclusion, this work describes the development of an alternative method based on hydrophilic ionic liquids for the preparation of genomic DNA from Gram-positive and Gram-negative bacterial cells. Compared to the often tedious and time-consuming protocols that are necessary for commercial kits or common enzymatic methods, the time, cost, and equipment for extracting DNA from bacteria is significantly reduced. We demonstrated that the DNA extracts can be readily used for (quantitative) PCR, and it can be speculated that they are also applicable to other molecular diagnostic methods such as DNA hybridization reactions, or DNA sequencing applications. Hence, the novel method is not limited to specialised diagnostics facilities, but it can be also applied in basic laboratories without molecular biological equipment. Furthermore, it would be conceivable to implement the method even in resource-limited settings and to combine it with tools for point-of-care diagnostics, e.g., isothermal DNA amplification methods^27^.

## Materials and Methods

### Ionic liquids used in this study

In total, eight ionic liquids were used for inhibition studies on qPCR as well as cell lysis experiments with *Enterococcus faecalis* as model organism (Table 1). Commercially available reagents and solvents for the synthesis of ionic liquids were used as received from Sigma-Aldrich (St. Louis, Missouri) unless otherwise specified. 1-Ethyl-3-methylimidazolium acetate, ([C_2_mim]OAc), 1-ethyl-3-methylimidazolium chloride ([C_2_mim]Cl), and choline dibutyl phosphate ([Cho]DBP) were purchased from Iolitec (Heilbronn, Germany) and used as received. Imidazolium-based ionic liquids 1-hexyl-3-methylimidazolium chloride ([C_6_mim]Cl) and 1-ethyl-3-methylimidazolium dimethylphosphate ([C_2_mim]Me_2_PO_4_) were synthesized according to known procedures and analytical data was in accordance with literature data^12,28^.

Choline based ionic liquids [Cho]Fmt, [Cho]Lac and [Cho]Hex, were prepared according to literature procedures, relying on the neutralization of freshly titrated commercially available choline bicarbonate solution with the corresponding acid in a ratio 1:0.95 to avoid the presence of any excess acid as exemplified on the synthesis of choline hexanoate:

A freshly titrated solution of choline bicarbonate (19.55 g, 90.80 mmol) was charged into a 3-necked round bottom flask and it was diluted with distilled water. Hexanoic acid (10.02 g, 86.26 mmol) was added dropwise to the reaction mixture. The reaction mixture was stirred at room temperature and concentrated *in vacuo*. Remaining solvent traces were removed under vacuum (0.2 mbar) with stirring for 20 hours at 40 °C. The product was obtained as a light yellowish gel (20.94 g, >99% yield). See Supplementary Materials for further IL synthesis descriptions and NMR data).

### Bacterial strains used in this study

The samples type for the investigations in this study consists of different microbiological cultures derived from plate or liquid cultivation. For this purpose, pure cultures of a total of eight bacterial type strains were used for the cell lysis experiments. Of these eight strains, four belong to the group of Gram-positive bacteria (*Enterococcus faecalis* NCTC 775, *Clostridium perfringens* NCTC 8237, *Bacillus subtilis* ATCC 6633, *Staphylococcus aureus* NCTC 6571) and four to the group of Gram-negative bacteria (*Escherichia coli* NCTC 9001, *Legionella pneumophila* NCTC 12821, *Pseudomonas aeruginosa* NCTC 10662, *Vibrio cholerae* ATCC 51352). For the screening experiments with the ionic liquids, *Enterococcus faecalis* NCTC 775 was cultivated for five hours at 37 °C in tryptic soy broth with yeast extract. The harvested liquid cultures were stored in 25% glycerol on –80 °C until further use. For the comparison of the five extraction methods, the cells of all eight strains were grown overnight on agar plates containing trypticase soy broth and yeast extract. They were subsequently suspended in Ringer’s solution for the cell count and extraction experiments. After the addition of glycerol to a final concentration of 25%, the cell suspensions were stored on −80 °C until further use.

### Total cell count by fluorescence microscopy

Dilutions of the bacterial cell suspensions were fixed with paraformaldehyde, filtered on polycarbonate filters, and stained with SYBR Gold for a subsequent total cell count under a Nikon Eclipse 80i fluorescence microscope.

### Preparation of bacterial suspensions for the subsequent extraction experiments

Aliquots of the liquid cultures or the suspensions were centrifuged for 5 min at 10,000 rpm, and the resulting cell pellets were washed twice and resuspended in the respective buffer that was used for the ionic liquid dilutions. Ten µl of the respective cell suspensions was used for each extraction procedure.

### DNA extraction procedures

#### Optimized DNA preparation procedure using IL/aqueous buffer systems

Ten µl of the pelleted and resuspended cells were mixed with 90 µl of the respective IL/buffer system (90% w/w [C_2_mim]OAc or 50% w/w [Cho]Hex in Tris pH 8 buffer) and incubated at 65 °C for 5 min. To overcome inhibitory effects caused by the ILs or cell components, the extract was diluted 1:20 with 10 mM Tris pH 8.0 before subsequent qPCR analyses (see Supplementary Fig. S14 for a schematic illustrating the workflow).

#### Extraction procedures using lysozyme/proteinase K with phenol/chloroform purification and commercial kits

Enzymatic DNA extraction with phenol/chloroform is a standard procedure in our laboratory^21^. Briefly, 10 µl of cell suspension of the respective strain in TE buffer was incubated twice for one hour at 37 °C after the additions of lysozyme and proteinase K, respectively. Following another 10 min incubation at 37 °C after adding sodium chloride and CTAB, the released DNA was separated from other cell components by the treatment with a combination of phenol and chloroform:isoamylalcohol in a 24:1 ratio. Finally, the DNA was precipitated using isopropanol, followed by a washing step with ethanol and the addition of 10 mM Tris pH 8.0 for resuspending the DNA pellet.

For the comparison of the extraction efficiencies with commercial kits, we used the QIAamp DNA Mini Kit from Qiagen and the Wizard Genomic DNA Purification Kit from Promega. The extraction procedures were carried out according to the manufacturers’ instructions for Gram-positive or Gram-negative bacteria, respectively.

### Quantification of bacterial DNA using quantitative PCR

#### *Enterococcus*-specific qPCR assay

To quantify the DNA in the inhibition and cell lysis experiments using *Enterococcus faecalis* as model organism, we applied a qPCR assay that specifically targets a region in the *Enterococcus* 23S rRNA gene (ENT-qPCR)^19,29^. The qPCR reactions were carried out in a total reaction volume of 15 µl containing 1 µM of each primer (MWG-Biotech AG, Ebersberg, Germany), 80 nmol L^−1^ of the probe (all oligonucleotide sequences are listed in Table 3), KAPA^TM^ Probe® Fast qPCR Master Mix 2 × (Peqlab, Erlangen, Germany), and 2.5 µl DNA extract. The reactions were performed on a 7500 Fast Real-Time PCR System (Applied Biosystems, New York, USA) according to the following protocol: 5 min at 95 °C, followed by 45 cycles of 15 s at 95 °C and 1 min at 60 °C. Unless noted otherwise, qPCR reactions were carried out in triplicate. The calibration curve was generated using a dilution series of DNA plasmid solution containing a known number of copies of the 23 S rRNA gene fragment targeted by the assay. No template controls (NTCs) were analysed in triplicate for every qPCR run. Data were only accepted when all NTCs were negative (see Supplementary Fig. S1 for an exemplary amplification plot and the respective standard curve).

**Table 3:** Oligonucleotides used in the qPCR reactions for quantifying the genomic DNA of the bacterial reference strains.

Assay	Oligonucleotide	Sequence (5′→3′)	References
ENT-qPCR	Forward	GAG AAA TTC CAA ACG AAC TTG (21)	USEPA^19^, Ludwig and Schleifer^29^
	Reverse	CAG TGC TCT ACC TCC ATC ATT (21)	
	Probe	TGG TTC TCT CCG AAA TAG CTT TAG GGC TA (29)	
16S-qPCR	8F	AGA GTT TGA TCC TGG CTC AG (20)	Frank *et al*.^31^
	338	CAT GCT GCC TCC CGT AGG AGT (21)	Fierer *et al*.^32^

#### Bacteria-specific qPCR assay

To quantify the DNA in the extraction experiment using eight different bacterial strains, we applied a qPCR assay that targets the V1-V2 region of the 16 S rRNA gene with primer binding sites that are universal to all bacteria (16S-qPCR)^30^. The qPCR reactions were carried out in a total reaction volume of 15 µl containing 200 nM of each primer (MWG-Biotech AG, Ebersberg, Germany; oligonucleotide sequences are listed in Tables 3), 12.5 µl KAPA™ SYBR® Fast qPCR Master Mix 2 × (Peqlab, Erlangen, Germany), 0.4 µg/µl BSA (Sigma-Aldrich, Vienna, Austria) and 2.5 µl DNA extract dilution. The reactions were performed on a 7500 Fast Real-Time PCR System (Applied Biosystems, New York, USA) according to the following protocol: 3 min at 95 °C, followed by 40 cycles of 30 s at 95 °C, 30 s at 57 °C, 1 min at 72 °C. Unless noted otherwise, qPCR reactions were carried out in duplicate. The calibration curve was generated using a dilution series of DNA plasmid solution containing a known number of copies of the 16 S rRNA gene fragment that is targeted by the assay. No template controls were analysed in triplicate for every qPCR run. Due to residual *Escherichia coli* DNA in the polymerase, small numbers of target copies are detected in each NTC. However, these numbers are several magnitudes lower than those in the actual samples. Hence, data were accepted when NTCs contained less than 100 copies of the 16S rRNA gene per reaction (see Supplementary Fig. S2 for an exemplary amplification plot and the respective standard curve).

### Statistical analysis

Data for the comparison of the extraction methods on different bacterial strains was subjected to statistical analysis. A repeated measures ANOVA with a Greenhouse-Geisser correction was used to compare mean results and highlight statistically significant differences between methods (for detailed results see Supplementary Material ANOVA). The phenol/chloroform method was defined as the reference method and pairwise comparisons to the reference were indicated in Fig. 5.

## Supplementary information

Supplementary Marerials

Supplementary Materials on Statistical Analysis

## Data Availability

The datasets generated during and/or analysed during the current study are available from the corresponding author on reasonable request.
