# Supplementary material for: Simple lysis of bacterial cells for DNA-based diagnostics using hydrophilic ionic liquids
Source: Sci Rep. 2019 Sep 30;9:13994. doi: 10.1038/s41598-019-50246-5 (PMC6768989; doi:10.1038/s41598-019-50246-5)
Supplement: Supplementary file 1 — Supplementary Marerials [file 41598_2019_50246_MOESM1_ESM.docx]

**Simple lysis of bacterial cells for DNA-based diagnostics using hydrophilic ionic liquids**

**Electronic Supplementary Material**

Roland Martzy^a,b^, Katharina Bica-Schröder^c^, Ádám Márk Pálvölgyi^c^, Claudia Kolm^a,b^, Stefan Jakwerth^b,d^, Alexander K. T. Kirschner^b,d,h^, Regina Sommer^b,d^, Rudolf Krska^e,f^, Robert L. Mach^g^, Andreas H. Farnleitner^b,h,i^, Georg H. Reischer^a,i^

^a^ TU Wien, Institute of Chemical, Environmental & Bioscience Engineering, Molecular Diagnostics Group, Department of Agrobiotechnology (IFA-Tulln), Tulln, Austria

^b^ ICC Interuniversity Cooperation Centre Water & Health, Vienna, Austria ([www.waterandhealth.at](http://www.waterandhealth.at))

^c^ TU Wien, Institute of Applied Synthetic Chemistry, Research Group for Sustainable Organic Synthesis and Catalysis, Vienna, Austria

^d^ Medical University Vienna, Institute for Hygiene and Applied Immunology, Unit Water Hygiene, Vienna, Austria

^e^ University of Natural Resources and Life Sciences Vienna (BOKU), Department of Agrobiotechnology (IFA-Tulln), Tulln, Austria

^f^ Institute for Global Food Security, School of Biological Sciences, Queen’s University Belfast, Northern Ireland, United Kingdom

^g^ TU Wien, Institute of Chemical, Environmental & Bioscience Engineering, Research Area Biochemical Technology 166/5, Vienna, Austria

^h^ Karl Landsteiner University of Health Sciences, Department for Pharmacology, Physiology and Microbiology, Research Area Water Quality and Health, Krems, Austria

^I^ TU Wien, Institute of Chemical, Environmental & Bioscience Engineering, Research Area Biochemical Technology, Research Group of Environmental Microbiology and Molecular Diagnostics, Vienna, Austria

[I. Exemplary amplification plots and standard curves 3](#_Toc14701074)

[II. Calculation of the limit of detection 5](#_Toc14701075)

[III. General remarks for the synthesis of ionic liquids 6](#_Toc14701076)

[IV. Synthesis and analytical data of ionic liquids 7](#_Toc14701077)

[V. NMR spectra of ionic liquids 10](#_Toc14701078)

[VI. Stability of choline hexanoate [Cho]Hex 15](#_Toc14701079)

[VII. Schematic workflow 17](#_Toc14701080)

[VIII. References 18](#_Toc14701081)

# Exemplary amplification plots and standard curves

**
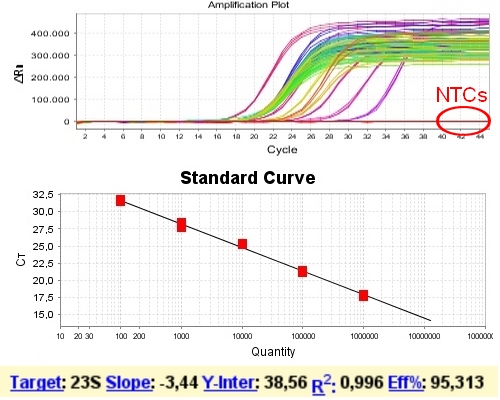
**

**Figure S1:** Exemplary amplification plot and respective standard curve derived from a USEPA *Enterococcus* spp. 23S rRNA gene qPCR run. The illustration corresponds to Figure 3 in the manuscript.


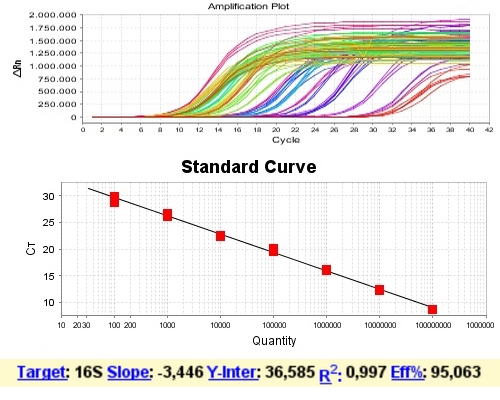


**Figure S2:** Exemplary amplification plot and respective standard curve derived from a bacteria-specific 16S rRNA gene qPCR run. The illustration corresponds to Figure 5 in the manuscript.

# II. Calculation of the limit of detection

**Limit of detection for the USEPA Enterococcus qPCR assay using the IL approach for DNA preparation:**

LOD_95%_ USEPA *Enterococcus* qPCR = 7 gene copies per 2.5 µl, i.e., per reaction ^1^

Considering the necessary dilution for the IL extracts (1:20) and assuming a cell lysis efficiency of 100%:

LOD_95%_ = 7 x 20 = 140 gene copies per 2.5 µl

Given that the *E. faecalis* 23S rRNA operon copy number is four ^2^ and assuming that all are detectable by qPCR:

140 gene copies / 4 = 35 *E. faecalis* cells in 2.5 µl

The entire extract has a volume of 100 µl (2.5 µl x 40):

35 cells x 40 = 1400 cells in 100 µl

These 1400 cells must be present in a cell suspension volume of 10 µl, since the remaining 90 µl are coming from the ILs. Hence, assuming a cell lysis efficiency of 100% the limit of detection (cells per ml) for the qPCR is:

LOD_95%_ = 1400 x 100 = 140,000 cells per ml suspension

# III. General remarks for the synthesis of ionic liquids

All reagents were purchased from commercial suppliers and used without purification unless noted otherwise.

1-Ethyl-3-methylimidazolium acetate ([C_2_mim]OAc), 1-ethyl-3-methylimidazolium chloride ([C_2_mim]Cl), and choline dibutyl phosphate ([Cho]Dbp) were purchased from *Iolitec* (Heilbronn, Germany) and used as received.

Imidazolium-based ionic liquid 1-hexyl-3-methylimidazolium chloride ([C_6_mim]Cl) and 1-ethyl-3-methylimidazolium dimethyl phosphate ([C_2_mim]Me_2_PO_4_) were synthesized according to known procedures by using freshly distilled *N-*methylimidazole and analytical data was in accordance with literature values ^3,4^. Choline based ionic liquids [Cho]Fmt, [Cho]Lac and [Cho]Hex were prepared according to literature procedures, relying on the neutralization of freshly titrated commercially available choline bicarbonate solution with the corresponding acid in a ratio 1:0.95 to avoid the presence of any excess acid. Analytical data was in accordance to literature values ^3,4^. All ionic liquids have been dried for 48 h at 25-40°C under high vacuum (<0.2 mBar) with stirring and were stored under argon before use.

For the characterization of the ionic liquids, ^1^H and ^13^C spectra were recorded from CDCl_3,_ D_2_O or DMSO-d_6_ solutions on a Bruker Advance UltraShield 400 (400 MHz) spectrometer and chemical shifts (δ) are reported in ppm, using tetramethylsilane as internal standard. Coupling constants (*J*) are reported in Hertz (Hz). The following abbreviations were used to explain the multiplicities: s = singlet, d = doublet, t = triplet, q = quartet, qvin. = qvintet, sex. = sextet, m = multiplet, brs = broad singlet.

# IV. Synthesis and analytical data of ionic liquids

## IV.1. Choline-based ionic liquids

*General procedure*

The exact concentration of choline bicarbonate (*Sigma Aldrich*, 80 w/w % solution) have been freshly determined *via* titration with 0.1 M HCl solution (volumetric) by using bromocresol-green as indicator before use. The ionic liquids have been prepared by dropwise addition of the corresponding acid to the base, using an appropriate solvent (methanol, water). After stirring the reaction mixtures at ambient temperature, the remaining volatiles have been removed under high vacuum (<0.2 mBar) to afford the corresponding products in quantitative yields.

*Choline hexanoate [Cho]Hex*

Light yellowish/colorless gel. ^1^H NMR (400 MHz, CDCl_3_) δ = 3.95 (br s, 2H, C*H_2_*-OH), 3.57 (t, *J* = 4.4 Hz, 2H, C*H_2_*-CH_2_-OH), 3.25 (s, 9H, 3 x N-C*H_3_*), 2.00 (t, *J* = 6.7 Hz, 2H, C*H_2_*-COO), 1.46 (qvin., *J* = 8.1 Hz, 2H, C*H_2_*-CH_2_-COO), 1.20 – 1.15 (m, 4H, C*H_2_*-(CH_2_)_2_-COO, C*H_2_*-(CH_2_)_3_-COO), 0.77 (t, *J* = 6.8 Hz, 3H, C*H_3_*-(CH_2_)_4_-COO). ^13^C NMR (100 MHz, D_2_O) δ = 183.96 (1C, *C*OO), 67.37 (1C, *C*H_2_-OH), 55.53 (1C, *C*H_2_-CH_2_-OH), 53.86 (3C, 3 x *C*H_3_), 37.59 (1C, *C*H_2_-COO), 31.00 (1C, *C*H_2_-CH_2_-COO), 25.56 (1C, *C*H_2_-(CH_2_)_2_-COO), 21.76 (1C, *C*H_2_-(CH_2_)_3_-COO), 13.29 (1C, *C*H_3_-(CH_2_)_4_-COO).

*Choline dibutyl phosphate [Cho]dbp (from Iolitec)*

Colorless solid. ^1^H NMR (400 MHz, DMSO-d_6_) δ = 3.81 (br s, 2H, C*H_2_*-OH), 3.58 (q, *J* = 4.0 Hz, 4H, 2 x C*H_2_*-O-P), 3.45 (t, *J* = 4.0 Hz, 2H, C*H_2_*-CH_2_-OH), 3.14 (s, 9H, 3 x N-C*H_3_*), 1.45 (qvin., *J* = 8.0 Hz, 2H, 2 x C*H_2_*-CH_2_-O-P), 1.30 (sex., *J* = 8.0 Hz, 2H, 2 x C*H_2_*-(CH_2_)_2_-O-P), 0.87 (t, *J* = 8.0 Hz, 6H, 2 x C*H_3_*-(CH_2_)_3_-O-P).

*Choline formate [Cho]Fmt*

Colorless oil. ^1^H NMR (400 MHz, DMSO-d_6_) δ = 8.52 (s, 1H, *H*COO), 3.94 (brs, 2H, C*H_2_*-OH), 3.44 (t, *J* = 4.0 Hz, 2H, C*H_2_*-CH_2_-OH), 3.14 (s, 9H, 3 x N-C*H_3_*).

*Choline lactate [Cho]Lac*

Colorless oil. ^1^H NMR (400 MHz, DMSO-d_6_) δ = 3.86 (brs, 2H, C*H_2_*-OH), 3.55 (q, *J* = 4.0 Hz, 2H, C*H*-COO), 3.44 (t, *J* = 4.0 Hz, 2H, C*H_2_*-CH_2_-OH), 3.14 (s, 9H, 3 x N-C*H_3_*), 1.09 (d, *J* = 8.0 Hz, 3H, C*H_3_*-CH-COO).

**IV.2. Imidazolium-based ionic liquids**

*1-Ethyl-3-methylimidazolium chloride [C_2_MIM]Cl*

Colorless solid. ^1^H NMR (400 MHz, CDCl_3_) δ = 10.09 (s, 1H, *H*-2), 7.58
(s, 2H, *H*-4, *H*-5), 4.31 (q, *J* = 8.0 Hz, 2H, N-C*H_2_*), 4.00 (s, 3H, N-C*H_3_*), 1.48 (t, *J* = 8.0 Hz, 2H, N-CH_2_-C*H_3_*).

*1-Hexyl-3-methylimidazolium chloride [C_6_MIM]Cl*

Colorless solid. ^1^H NMR (400 MHz, CDCl_3_) δ = 10.46 (s, 1H, *H*-2), 7.62 (t, *J* = 4.0 Hz, 1H, *H*-4), 7.40 (t, *J* = 4.0 Hz, 1H, *H*-5), 4.21 (t, *J* = 4.0 Hz, 2H, N-C*H_2_*), 4.02 (s, 3H, N-C*H_3_*), 1.80 (qvin., *J* = 8.0 Hz, 2H, N-CH_2_-C*H_2_*), 1.24 – 1.16 (m, 6H, N-(CH_2_)_2_-(C*H_2_*)*_3_*-CH_3_), 0.75 (t, *J* = 8.0 Hz, 3H, N-(CH_2_)_5_-C*H_3_*).

*1-Ethyl-3-methylimidazolium dimethyl phosphate [C_2_MIM]Me_2_PO_4_*

Colorless oil. ^1^H NMR (400 MHz, DMSO-d_6_) δ = 9.68 (s, 1H, *H*-2), 7.93 (t, *J* = 4.0 Hz, 1H, *H*-4), 7.83 (t, *J* = 4.0 Hz, 1H, *H*-5), 4.23 (q, *J* = 8.0 Hz, 2H, N-C*H_2_*), 3.88 (s, 3H, N-C*H_3_*), 3.29 (d, *J* = 8.0 Hz, 6H, 2 x P-O-C*H_3_*), 1.40 (t, *J* = 8.0 Hz, 3H, N-CH_2_-C*H_3_*).

*1-Ethyl-3-methylimidazolium acetate [C_2_MIM]OAc*

Colorless oil. ^1^H NMR (400 MHz, CDCl_3_) δ = 10.16 (s, 1H, *H*-2), 7.94 (t, *J* = 4.0 Hz, 1H, *H*-4), 7.84 (t, *J* = 4.0 Hz, 1H, *H*-5), 4.23 (q, *J* = 8.0 Hz, 2H, N-C*H_2_*), 3.89 (s, 3H, N-C*H_3_*), 1.60 (s, 3H, C*H_3_-*COO), 1.40 (t, *J* = 8.0 Hz, 3H, N-CH_2_-C*H_3_*).

# V. NMR spectra of ionic liquids

**V.1. Choline hexanoate [Cho]Hex**

** **Figure S3:** ^1^H NMR spectrum of [Cho]Hex

**Figure S4:** ^13^C APT NMR spectrum of [Cho]Hex

**V.2. Choline dibutyl phosphate [Cho]Dbp**

**Figure S5:** ^1^H NMR spectrum of [Cho]Dbp

**V.3. Choline formate [Cho]Fmt**

***Figure S6:*** *^1^H NMR spectrum of [Cho]Fmt*

**V.4. Choline lactate [Cho]Lac**

**Figure S7:** ^1^H NMR spectrum of [Cho]Lac

**V.5. 1-Etyl-3-methylimidazolium chloride [C_2_MIM]Cl**

**Figure S8:** ^1^H NMR spectrum of [C_2_MIM]Cl

**V.6. 1-Hexyl-3-methylimidazolium chloride [C_6_MIM]Cl**

**Figure S9:** ^1^H NMR spectrum of [C_6_MIM]Cl

**V.7. 1-Ethyl-3-methylimidazolium dimethyl phosphate [C_2_MIM]Me_2_PO_4_**

**Figure S10:** ^1^H NMR spectrum of [C_2_MIM]Me_2_PO_4_

**V.8. 1-Ethyl-3-methylimidazolium acetate [C_2_MIM]OAc**

**Figure S11:** ^1^H NMR spectrum of [C_2_MIM]OAc

# VI. Stability of choline hexanoate [Cho]Hex

In order to investigate the stability of [Cho]Hex, the ^1^H NMR and ^13^C NMR spectra of two samples have been compared:

(A): Freshly prepared and dried ionic liquid

(B): A 16 months old sample, stored at room temperature on a bench shelf

Based on the results from NMR spectroscopy (Figure S12 and S13), no significant change in the purity of the ionic liquid was observed, indicating excellent stability during storage at ambient conditions.

**Figure S12:** ^1^H NMR spectra of the freshly prepared [Cho]Hex (**A**) and a 16 months old sample (**B**)

**Figure S13:** ^13^C APT NMR spectra of the freshly prepared [Cho]Hex (**A**) a 16 months old sample (**B**)

# VII. Schematic workflow


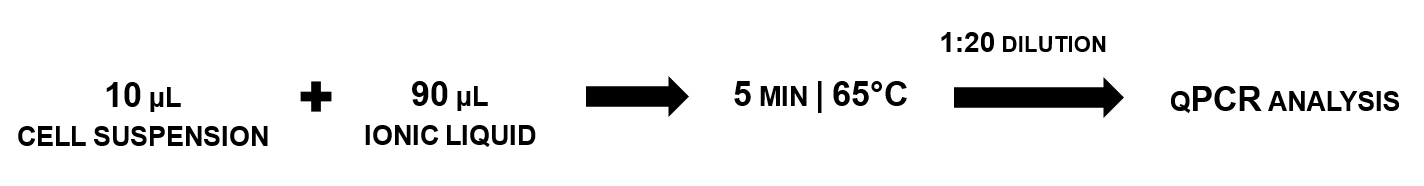
Figure S14: Schematic illustrating the simple workflow for the lysis of bacterial cells using hydrophilic ionic liquids, followed by qPCR analysis.

# VIII. References

1 Martzy, R. *et al.* A loop-mediated isothermal amplification (LAMP) assay for the rapid detection of Enterococcus spp. in water. *Water Research* **122**, 62-69, doi:10.1016/j.watres.2017.05.023 (2017).

2 Marshall, S. H., Donskey, C. J., Hutton-Thomas, R., Salata, R. A. & Rice, L. B. Gene dosage and linezolid resistance in Enterococcus faecium and Enterococcus faecalis. *Antimicrobial agents and chemotherapy* **46**, 3334-3336 (2002).

3 Kuhlmann, E., Himmler, S., Giebelhaus, H. & Wasserscheid, P. Imidazolium dialkylphosphates—a class of versatile, halogen-free and hydrolytically stable ionic liquids. *Green Chemistry* **9**, 233-242, doi:10.1039/B611974C (2007).

4 Gonzalez-García, E. *et al.* Direct extraction of genomic DNA from maize with aqueous ionic liquid buffer systems for applications in genetically modified organisms analysis. *Analytical and Bioanalytical Chemistry* **406**, 7773-7784, doi:10.1007/s00216-014-8204-y (2014).
