# Supplementary material for: Simple lysis of bacterial cells for DNA-based diagnostics using hydrophilic ionic liquids
Source: Sci Rep. 2019 Sep 30;9:13994. doi: 10.1038/s41598-019-50246-5 (PMC6768989; doi:10.1038/s41598-019-50246-5)

**Simple lysis of bacterial cells for DNA-based diagnostics using hydrophilic ionic liquids**

**Electronic Supplementary Material**

Roland Martzy^a,b^, Katharina Bica-Schröder^c^, Ádám Márk Pálvölgyi^c^, Claudia Kolm^a,b^, Stefan Jakwerth^b,d^, Alexander K. T. Kirschner^b,d,h^, Regina Sommer^b,d^, Rudolf Krska^e,f^, Robert L. Mach^g^, Andreas H. Farnleitner^b,h,i^, Georg H. Reische^a,i^

^a^ TU Wien, Institute of Chemical, Environmental & Bioscience Engineering, Molecular Diagnostics Group, Department of Agrobiotechnology (IFA-Tulln), Tulln, Austria

^b^ ICC Interuniversity Cooperation Centre Water & Health, Vienna, Austria ([www.waterandhealth.at](http://www.waterandhealth.at))

^c^ TU Wien, Institute of Applied Synthetic Chemistry, Research Group for Sustainable Organic Synthesis and Catalysis, Vienna, Austria

^d^ Medical University Vienna, Institute for Hygiene and Applied Immunology, Unit Water Hygiene, Vienna, Austria

^e^ University of Natural Resources and Life Sciences Vienna (BOKU), Department of Agrobiotechnology (IFA-Tulln), Tulln, Austria

^f^ Institute for Global Food Security, School of Biological Sciences, Queen’s University Belfast, Northern Ireland, United Kingdom

^g^ TU Wien, Institute of Chemical, Environmental & Bioscience Engineering, Research Area Biochemical Technology 166/5, Vienna, Austria

^h^ Karl Landsteiner University of Health Sciences, Department for Pharmacology, Physiology and Microbiology, Research Area Water Quality and Health, Krems, Austria

^I^ TU Wien, Institute of Chemical, Environmental & Bioscience Engineering, Research Area Biochemical Technology, Research Group of Environmental Microbiology and Molecular Diagnostics, Vienna, Austria

**Statistical analysis of extraction method comparison using ANOVA with repeated measurements**

**General Linear Model**

| **Notes** | | |
| --- | --- | --- |
| Output Created | | 22-JUL-2019 09:04:29 |
| Comments | |  |
| Input | Data |  |
|  | Active Dataset | DataSet1 |
|  | Filter | <none> |
|  | Weight | <none> |
|  | Split File | Strain |
|  | N of Rows in Working Data File | 96 |
| Missing Value Handling | Definition of Missing | User-defined missing values are treated as missing. |
|  | Cases Used | Statistics are based on all cases with valid data for all variables in the model. |
| Syntax | | GLM PheChl Promega Qiagen C2mimOAc ChoHex  /WSFACTOR=Extraction_method 5 Polynomial  /METHOD=SSTYPE(3)  /PLOT=PROFILE(Extraction_method) TYPE=BAR ERRORBAR=CI MEANREFERENCE=NO  /EMMEANS=TABLES(Extraction_method) COMPARE ADJ(BONFERRONI)  /PRINT=DESCRIPTIVE ETASQ  /CRITERIA=ALPHA(.05)  /WSDESIGN=Extraction_method. |
| Resources | Processor Time | 00:00:02,82 |
|  | Elapsed Time | 00:00:05,00 |

|  |
| --- |

| Measure: MEASURE_1 | |
| --- | --- |
| Extraction_method | Dependent Variable |
| 1 | PheChl |
| 2 | Promega |
| 3 | Qiagen |
| 4 | C2mimOAc |
| 5 | ChoHex |

**Strain = B. subtilis**

| a. Strain = B. subtilis |
| --- |

| **Descriptive Statistics^a^** | | | |
| --- | --- | --- | --- |
|  | Mean | Std. Deviation | N |
| PheChl | 8.7058 | .05854 | 12 |
| Promega | 5.4125 | .47517 | 12 |
| Qiagen | 8.2825 | .15621 | 12 |
| C2mimOAc | 6.6175 | .16477 | 12 |
| ChoHex | 7.1225 | .18430 | 12 |

| a. Strain = B. subtilis |
| --- |

| **Multivariate Tests^a,b^** | | | | | | | |
| --- | --- | --- | --- | --- | --- | --- | --- |
| Effect | | Value | F | Hypothesis df | Error df | Sig. | Partial Eta Squared |
| Extraction_method | Pillai's Trace | 1.000 | 5956.648^c^ | 4.000 | 8.000 | .000 | 1.000 |
|  | Wilks' Lambda | .000 | 5956.648^c^ | 4.000 | 8.000 | .000 | 1.000 |
|  | Hotelling's Trace | 2978.324 | 5956.648^c^ | 4.000 | 8.000 | .000 | 1.000 |
|  | Roy's Largest Root | 2978.324 | 5956.648^c^ | 4.000 | 8.000 | .000 | 1.000 |

| a. Strain = B. subtilis |
| --- |
| b. Design: Intercept  Within Subjects Design: Extraction_method |
| c. Exact statistic |

| **Mauchly's Test of Sphericity^a,b^** | | | | | | | |
| --- | --- | --- | --- | --- | --- | --- | --- |
| Measure: MEASURE_1 | | | | | | | |
| Within Subjects Effect | Mauchly's W | Approx. Chi-Square | df | Sig. | Epsilon^c^ | |  |
|  |  |  |  |  | Greenhouse-Geisser | Huynh-Feldt |  |
| Extraction_method | .001 | 71.462 | 9 | .000 | .280 | .290 |  |

| **Tests of Within-Subjects Effects^a^** | | | | | | | |
| --- | --- | --- | --- | --- | --- | --- | --- |
| Measure: MEASURE_1 | | | | | | | |
| Source | | Type III Sum of Squares | df | Mean Square | F | Sig. |  |
| Extraction_method | Sphericity Assumed | 83.710 | 4 | 20.928 | 277.095 | .000 |  |
|  | Greenhouse-Geisser | 83.710 | 1.121 | 74.655 | 277.095 | .000 |  |
|  | Huynh-Feldt | 83.710 | 1.160 | 72.188 | 277.095 | .000 |  |
|  | Lower-bound | 83.710 | 1.000 | 83.710 | 277.095 | .000 |  |
| Error(Extraction_method) | Sphericity Assumed | 3.323 | 44 | .076 |  |  |  |
|  | Greenhouse-Geisser | 3.323 | 12.334 | .269 |  |  |  |
|  | Huynh-Feldt | 3.323 | 12.756 | .261 |  |  |  |
|  | Lower-bound | 3.323 | 11.000 | .302 |  |  |  |

| **Tests of Within-Subjects Contrasts^a^** | | | | | | | |
| --- | --- | --- | --- | --- | --- | --- | --- |
| Measure: MEASURE_1 | | | | | | | |
| Source | Extraction_method | Type III Sum of Squares | df | Mean Square | F | Sig. |  |
| Extraction_method | Linear | 4.618 | 1 | 4.618 | 48.615 | .000 |  |
|  | Quadratic | 8.035 | 1 | 8.035 | 417.264 | .000 |  |
|  | Cubic | 19.136 | 1 | 19.136 | 156.710 | .000 |  |
|  | Order 4 | 51.922 | 1 | 51.922 | 789.739 | .000 |  |
| Error(Extraction_method) | Linear | 1.045 | 11 | .095 |  |  |  |
|  | Quadratic | .212 | 11 | .019 |  |  |  |
|  | Cubic | 1.343 | 11 | .122 |  |  |  |
|  | Order 4 | .723 | 11 | .066 |  |  |  |

| **Tests of Between-Subjects Effects^a^** | | | | | | |
| --- | --- | --- | --- | --- | --- | --- |
| Measure: MEASURE_1 | | | | | | |
| Transformed Variable: Average | | | | | | |
| Source | Type III Sum of Squares | df | Mean Square | F | Sig. | Partial Eta Squared |
| Intercept | 3134.784 | 1 | 3134.784 | 248257.980 | .000 | 1.000 |
| Error | .139 | 11 | .013 |  |  |  |

| a. Strain = B. subtilis |
| --- |

**Estimated Marginal Means**

**Extraction_method**

| **Estimates^a^** | | | | |
| --- | --- | --- | --- | --- |
| Measure: MEASURE_1 | | | | |
| Extraction_method | Mean | Std. Error | 95% Confidence Interval | |
|  |  |  | Lower Bound | Upper Bound |
| 1 | 8.706 | .017 | 8.669 | 8.743 |
| 2 | 5.413 | .137 | 5.111 | 5.714 |
| 3 | 8.283 | .045 | 8.183 | 8.382 |
| 4 | 6.618 | .048 | 6.513 | 6.722 |
| 5 | 7.122 | .053 | 7.005 | 7.240 |
| a. Strain = B. subtilis | | | | |

| **Pairwise Comparisons^a^** | | | | | | |
| --- | --- | --- | --- | --- | --- | --- |
| Measure: MEASURE_1 | | | | | | |
| (I) Extraction_method | (J) Extraction_method | Mean Difference (I-J) | Std. Error | Sig.^c^ | 95% Confidence Interval for Difference^c^ | |
|  |  |  |  |  | Lower Bound | Upper Bound |
| 1 | 2 | 3.293^*^ | .133 | .000 | 2.827 | 3.760 |
|  | 3 | .423^*^ | .053 | .000 | .237 | .609 |
|  | 4 | 2.088^*^ | .054 | .000 | 1.901 | 2.276 |
|  | 5 | 1.583^*^ | .052 | .000 | 1.402 | 1.765 |
| 2 | 1 | -3.293^*^ | .133 | .000 | -3.760 | -2.827 |
|  | 3 | -2.870^*^ | .171 | .000 | -3.469 | -2.271 |
|  | 4 | -1.205^*^ | .183 | .000 | -1.845 | -.565 |
|  | 5 | -1.710^*^ | .182 | .000 | -2.345 | -1.075 |
| 3 | 1 | -.423^*^ | .053 | .000 | -.609 | -.237 |
|  | 2 | 2.870^*^ | .171 | .000 | 2.271 | 3.469 |
|  | 4 | 1.665^*^ | .035 | .000 | 1.544 | 1.786 |
|  | 5 | 1.160^*^ | .044 | .000 | 1.005 | 1.315 |
| 4 | 1 | -2.088^*^ | .054 | .000 | -2.276 | -1.901 |
|  | 2 | 1.205^*^ | .183 | .000 | .565 | 1.845 |
|  | 3 | -1.665^*^ | .035 | .000 | -1.786 | -1.544 |
|  | 5 | -.505^*^ | .025 | .000 | -.594 | -.416 |
| 5 | 1 | -1.583^*^ | .052 | .000 | -1.765 | -1.402 |
|  | 2 | 1.710^*^ | .182 | .000 | 1.075 | 2.345 |
|  | 3 | -1.160^*^ | .044 | .000 | -1.315 | -1.005 |
|  | 4 | .505^*^ | .025 | .000 | .416 | .594 |

| Based on estimated marginal means^a^ |
| --- |
| *. The mean difference is significant at the .05 level. |
| a. Strain = B. subtilis |
| c. Adjustment for multiple comparisons: Bonferroni. |

| **Multivariate Tests^a^** | | | | | | |
| --- | --- | --- | --- | --- | --- | --- |
|  | Value | F | Hypothesis df | Error df | Sig. | Partial Eta Squared |
| Pillai's trace | 1.000 | 5956.648^b^ | 4.000 | 8.000 | .000 | 1.000 |
| Wilks' lambda | .000 | 5956.648^b^ | 4.000 | 8.000 | .000 | 1.000 |
| Hotelling's trace | 2978.324 | 5956.648^b^ | 4.000 | 8.000 | .000 | 1.000 |
| Roy's largest root | 2978.324 | 5956.648^b^ | 4.000 | 8.000 | .000 | 1.000 |

| Each F tests the multivariate effect of Extraction_method. These tests are based on the linearly independent pairwise comparisons among the estimated marginal means.^a^ |
| --- |
| a. Strain = B. subtilis |
| b. Exact statistic |

**Profile Plots**


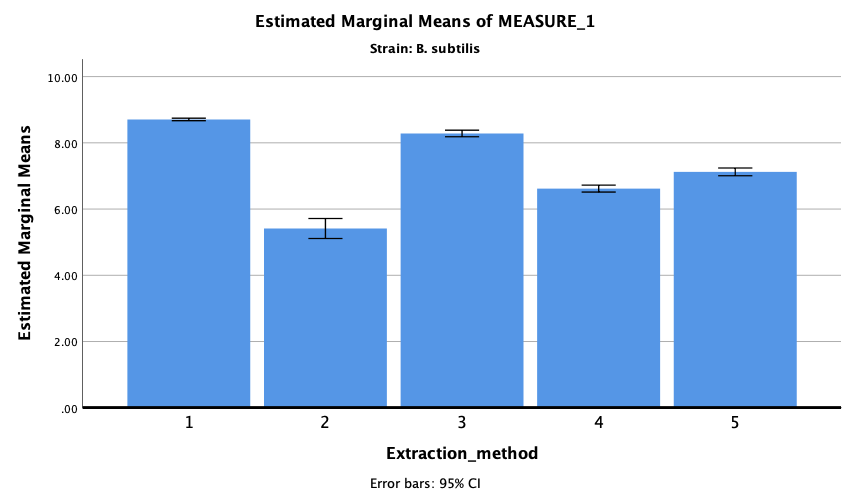


**Strain = C. perfringens**

| **Descriptive Statistics^a^** | | | |
| --- | --- | --- | --- |
|  | Mean | Std. Deviation | N |
| PheChl | 9.5683 | .04569 | 12 |
| Promega | 6.7875 | .61304 | 12 |
| Qiagen | 9.5442 | .07217 | 12 |
| C2mimOAc | 8.9075 | .17221 | 12 |
| ChoHex | 9.1883 | .30262 | 12 |

| a. Strain = C. perfringens |
| --- |

| **Multivariate Tests^a,b^** | | | | | | | |
| --- | --- | --- | --- | --- | --- | --- | --- |
| Effect | | Value | F | Hypothesis df | Error df | Sig. | Partial Eta Squared |
| Extraction_method | Pillai's Trace | .977 | 86.710^c^ | 4.000 | 8.000 | .000 | .977 |
|  | Wilks' Lambda | .023 | 86.710^c^ | 4.000 | 8.000 | .000 | .977 |
|  | Hotelling's Trace | 43.355 | 86.710^c^ | 4.000 | 8.000 | .000 | .977 |
|  | Roy's Largest Root | 43.355 | 86.710^c^ | 4.000 | 8.000 | .000 | .977 |

| a. Strain = C. perfringens |
| --- |
| b. Design: Intercept  Within Subjects Design: Extraction_method |
| c. Exact statistic |

| **Mauchly's Test of Sphericity^a,b^** | | | | | | | |
| --- | --- | --- | --- | --- | --- | --- | --- |
| Measure: MEASURE_1 | | | | | | | |
| Within Subjects Effect | Mauchly's W | Approx. Chi-Square | df | Sig. | Epsilon^c^ | |  |
|  |  |  |  |  | Greenhouse-Geisser | Huynh-Feldt |  |
| Extraction_method | .001 | 64.258 | 9 | .000 | .372 | .417 |  |

| **Tests of Within-Subjects Effects^a^** | | | | | | | |
| --- | --- | --- | --- | --- | --- | --- | --- |
| Measure: MEASURE_1 | | | | | | | |
| Source | | Type III Sum of Squares | df | Mean Square | F | Sig. |  |
| Extraction_method | Sphericity Assumed | 64.280 | 4 | 16.070 | 162.190 | .000 |  |
|  | Greenhouse-Geisser | 64.280 | 1.488 | 43.204 | 162.190 | .000 |  |
|  | Huynh-Feldt | 64.280 | 1.667 | 38.568 | 162.190 | .000 |  |
|  | Lower-bound | 64.280 | 1.000 | 64.280 | 162.190 | .000 |  |
| Error(Extraction_method) | Sphericity Assumed | 4.360 | 44 | .099 |  |  |  |
|  | Greenhouse-Geisser | 4.360 | 16.366 | .266 |  |  |  |
|  | Huynh-Feldt | 4.360 | 18.333 | .238 |  |  |  |
|  | Lower-bound | 4.360 | 11.000 | .396 |  |  |  |

| **Tests of Within-Subjects Contrasts^a^** | | | | | | | |
| --- | --- | --- | --- | --- | --- | --- | --- |
| Measure: MEASURE_1 | | | | | | | |
| Source | Extraction_method | Type III Sum of Squares | df | Mean Square | F | Sig. |  |
| Extraction_method | Linear | 2.220 | 1 | 2.220 | 36.299 | .000 |  |
|  | Quadratic | 6.388 | 1 | 6.388 | 91.815 | .000 |  |
|  | Cubic | 25.613 | 1 | 25.613 | 233.678 | .000 |  |
|  | Order 4 | 30.059 | 1 | 30.059 | 192.695 | .000 |  |
| Error(Extraction_method) | Linear | .673 | 11 | .061 |  |  |  |
|  | Quadratic | .765 | 11 | .070 |  |  |  |
|  | Cubic | 1.206 | 11 | .110 |  |  |  |
|  | Order 4 | 1.716 | 11 | .156 |  |  |  |

| **Tests of Between-Subjects Effects^a^** | | | | | | |
| --- | --- | --- | --- | --- | --- | --- |
| Measure: MEASURE_1 | | | | | | |
| Transformed Variable: Average | | | | | | |
| Source | Type III Sum of Squares | df | Mean Square | F | Sig. | Partial Eta Squared |
| Intercept | 4645.520 | 1 | 4645.520 | 43001.828 | .000 | 1.000 |
| Error | 1.188 | 11 | .108 |  |  |  |

| a. Strain = C. perfringens |
| --- |

**Estimated Marginal Means**

**Extraction_method**

| **Estimates^a^** | | | | |
| --- | --- | --- | --- | --- |
| Measure: MEASURE_1 | | | | |
| Extraction_method | Mean | Std. Error | 95% Confidence Interval | |
|  |  |  | Lower Bound | Upper Bound |
| 1 | 9.568 | .013 | 9.539 | 9.597 |
| 2 | 6.788 | .177 | 6.398 | 7.177 |
| 3 | 9.544 | .021 | 9.498 | 9.590 |
| 4 | 8.907 | .050 | 8.798 | 9.017 |
| 5 | 9.188 | .087 | 8.996 | 9.381 |
| a. Strain = C. perfringens | | | | |

| **Pairwise Comparisons^a^** | | | | | | |
| --- | --- | --- | --- | --- | --- | --- |
| Measure: MEASURE_1 | | | | | | |
| (I) Extraction_method | (J) Extraction_method | Mean Difference (I-J) | Std. Error | Sig.^c^ | 95% Confidence Interval for Difference^c^ | |
|  |  |  |  |  | Lower Bound | Upper Bound |
| 1 | 2 | 2.781^*^ | .181 | .000 | 2.149 | 3.412 |
|  | 3 | .024 | .013 | .936 | -.022 | .070 |
|  | 4 | .661^*^ | .055 | .000 | .468 | .853 |
|  | 5 | .380^*^ | .085 | .009 | .083 | .677 |
| 2 | 1 | -2.781^*^ | .181 | .000 | -3.412 | -2.149 |
|  | 3 | -2.757^*^ | .187 | .000 | -3.410 | -2.104 |
|  | 4 | -2.120^*^ | .146 | .000 | -2.630 | -1.610 |
|  | 5 | -2.401^*^ | .206 | .000 | -3.120 | -1.681 |
| 3 | 1 | -.024 | .013 | .936 | -.070 | .022 |
|  | 2 | 2.757^*^ | .187 | .000 | 2.104 | 3.410 |
|  | 4 | .637^*^ | .065 | .000 | .408 | .865 |
|  | 5 | .356^*^ | .084 | .014 | .063 | .649 |
| 4 | 1 | -.661^*^ | .055 | .000 | -.853 | -.468 |
|  | 2 | 2.120^*^ | .146 | .000 | 1.610 | 2.630 |
|  | 3 | -.637^*^ | .065 | .000 | -.865 | -.408 |
|  | 5 | -.281 | .111 | .280 | -.669 | .108 |
| 5 | 1 | -.380^*^ | .085 | .009 | -.677 | -.083 |
|  | 2 | 2.401^*^ | .206 | .000 | 1.681 | 3.120 |
|  | 3 | -.356^*^ | .084 | .014 | -.649 | -.063 |
|  | 4 | .281 | .111 | .280 | -.108 | .669 |

| Based on estimated marginal means^a^ |
| --- |
| *. The mean difference is significant at the .05 level. |
| a. Strain = C. perfringens |
| c. Adjustment for multiple comparisons: Bonferroni. |

| **Multivariate Tests^a^** | | | | | | |
| --- | --- | --- | --- | --- | --- | --- |
|  | Value | F | Hypothesis df | Error df | Sig. | Partial Eta Squared |
| Pillai's trace | .977 | 86.710^b^ | 4.000 | 8.000 | .000 | .977 |
| Wilks' lambda | .023 | 86.710^b^ | 4.000 | 8.000 | .000 | .977 |
| Hotelling's trace | 43.355 | 86.710^b^ | 4.000 | 8.000 | .000 | .977 |
| Roy's largest root | 43.355 | 86.710^b^ | 4.000 | 8.000 | .000 | .977 |

| Each F tests the multivariate effect of Extraction_method. These tests are based on the linearly independent pairwise comparisons among the estimated marginal means.^a^ |
| --- |
| a. Strain = C. perfringens |
| b. Exact statistic |

**Profile Plots**


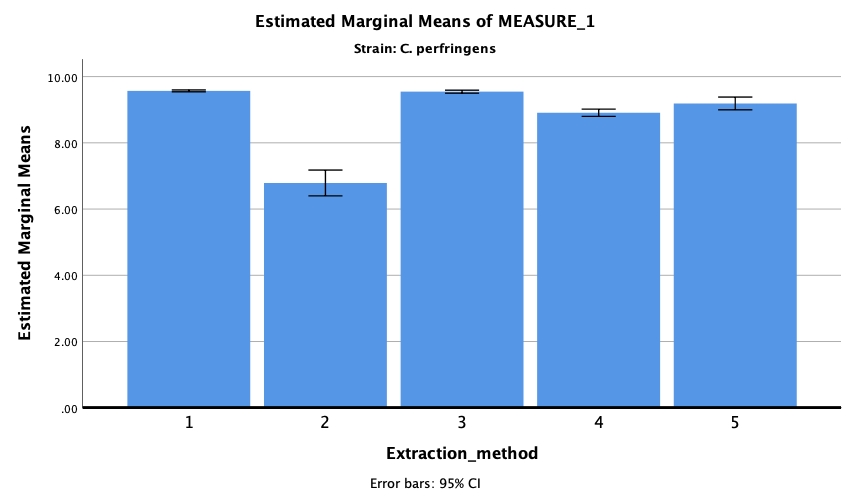


**Strain = E. coli**

| **Descriptive Statistics^a^** | | | |
| --- | --- | --- | --- |
|  | Mean | Std. Deviation | N |
| PheChl | 9.2092 | .04870 | 12 |
| Promega | 6.3625 | .35173 | 12 |
| Qiagen | 9.2192 | .10808 | 12 |
| C2mimOAc | 8.7017 | .07849 | 12 |
| ChoHex | 8.7642 | .18466 | 12 |

| a. Strain = E. coli |
| --- |

| **Multivariate Tests^a,b^** | | | | | | | |
| --- | --- | --- | --- | --- | --- | --- | --- |
| Effect | | Value | F | Hypothesis df | Error df | Sig. | Partial Eta Squared |
| Extraction_method | Pillai's Trace | .993 | 305.268^c^ | 4.000 | 8.000 | .000 | .993 |
|  | Wilks' Lambda | .007 | 305.268^c^ | 4.000 | 8.000 | .000 | .993 |
|  | Hotelling's Trace | 152.634 | 305.268^c^ | 4.000 | 8.000 | .000 | .993 |
|  | Roy's Largest Root | 152.634 | 305.268^c^ | 4.000 | 8.000 | .000 | .993 |
| a. Strain = E. coli | | | | | | | |
| b. Design: Intercept  Within Subjects Design: Extraction_method | | | | | | | |
| c. Exact statistic | | | | | | | |

| **Mauchly's Test of Sphericity^a,b^** | | | | | | | |
| --- | --- | --- | --- | --- | --- | --- | --- |
| Measure: MEASURE_1 | | | | | | | |
| Within Subjects Effect | Mauchly's W | Approx. Chi-Square | df | Sig. | Epsilon^c^ | |  |
|  |  |  |  |  | Greenhouse-Geisser | Huynh-Feldt |  |
| Extraction_method | .008 | 46.026 | 9 | .000 | .417 | .483 |  |

| **Tests of Within-Subjects Effects^a^** | | | | | | | |
| --- | --- | --- | --- | --- | --- | --- | --- |
| Measure: MEASURE_1 | | | | | | | |
| Source | | Type III Sum of Squares | df | Mean Square | F | Sig. |  |
| Extraction_method | Sphericity Assumed | 68.252 | 4 | 17.063 | 531.038 | .000 |  |
|  | Greenhouse-Geisser | 68.252 | 1.669 | 40.885 | 531.038 | .000 |  |
|  | Huynh-Feldt | 68.252 | 1.933 | 35.316 | 531.038 | .000 |  |
|  | Lower-bound | 68.252 | 1.000 | 68.252 | 531.038 | .000 |  |
| Error(Extraction_method) | Sphericity Assumed | 1.414 | 44 | .032 |  |  |  |
|  | Greenhouse-Geisser | 1.414 | 18.363 | .077 |  |  |  |
|  | Huynh-Feldt | 1.414 | 21.258 | .067 |  |  |  |
|  | Lower-bound | 1.414 | 11.000 | .129 |  |  |  |

| **Tests of Within-Subjects Contrasts^a^** | | | | | | | |
| --- | --- | --- | --- | --- | --- | --- | --- |
| Measure: MEASURE_1 | | | | | | | |
| Source | Extraction_method | Type III Sum of Squares | df | Mean Square | F | Sig. |  |
| Extraction_method | Linear | 2.520 | 1 | 2.520 | 138.891 | .000 |  |
|  | Quadratic | 5.121 | 1 | 5.121 | 392.653 | .000 |  |
|  | Cubic | 31.498 | 1 | 31.498 | 512.237 | .000 |  |
|  | Order 4 | 29.113 | 1 | 29.113 | 812.116 | .000 |  |
| Error(Extraction_method) | Linear | .200 | 11 | .018 |  |  |  |
|  | Quadratic | .143 | 11 | .013 |  |  |  |
|  | Cubic | .676 | 11 | .061 |  |  |  |
|  | Order 4 | .394 | 11 | .036 |  |  |  |

| **Tests of Between-Subjects Effects^a^** | | | | | | |
| --- | --- | --- | --- | --- | --- | --- |
| Measure: MEASURE_1 | | | | | | |
| Transformed Variable: Average | | | | | | |
| Source | Type III Sum of Squares | df | Mean Square | F | Sig. | Partial Eta Squared |
| Intercept | 4285.502 | 1 | 4285.502 | 86576.860 | .000 | 1.000 |
| Error | .544 | 11 | .049 |  |  |  |

| a. Strain = E. coli |
| --- |

**Estimated Marginal Means**

**Extraction_method**

| **Estimates^a^** | | | | |
| --- | --- | --- | --- | --- |
| Measure: MEASURE_1 | | | | |
| Extraction_method | Mean | Std. Error | 95% Confidence Interval | |
|  |  |  | Lower Bound | Upper Bound |
| 1 | 9.209 | .014 | 9.178 | 9.240 |
| 2 | 6.362 | .102 | 6.139 | 6.586 |
| 3 | 9.219 | .031 | 9.150 | 9.288 |
| 4 | 8.702 | .023 | 8.652 | 8.752 |
| 5 | 8.764 | .053 | 8.647 | 8.881 |
| a. Strain = E. coli | | | | |

| **Pairwise Comparisons^a^** | | | | | | |
| --- | --- | --- | --- | --- | --- | --- |
| Measure: MEASURE_1 | | | | | | |
| (I) Extraction_method | (J) Extraction_method | Mean Difference (I-J) | Std. Error | Sig.^c^ | 95% Confidence Interval for Difference^c^ | |
|  |  |  |  |  | Lower Bound | Upper Bound |
| 1 | 2 | 2.847^*^ | .103 | .000 | 2.488 | 3.206 |
|  | 3 | -.010 | .023 | 1.000 | -.091 | .071 |
|  | 4 | .508^*^ | .027 | .000 | .412 | .603 |
|  | 5 | .445^*^ | .044 | .000 | .290 | .600 |
| 2 | 1 | -2.847^*^ | .103 | .000 | -3.206 | -2.488 |
|  | 3 | -2.857^*^ | .102 | .000 | -3.215 | -2.498 |
|  | 4 | -2.339^*^ | .106 | .000 | -2.711 | -1.968 |
|  | 5 | -2.402^*^ | .103 | .000 | -2.762 | -2.042 |
| 3 | 1 | .010 | .023 | 1.000 | -.071 | .091 |
|  | 2 | 2.857^*^ | .102 | .000 | 2.498 | 3.215 |
|  | 4 | .518^*^ | .049 | .000 | .347 | .688 |
|  | 5 | .455^*^ | .046 | .000 | .294 | .616 |
| 4 | 1 | -.508^*^ | .027 | .000 | -.603 | -.412 |
|  | 2 | 2.339^*^ | .106 | .000 | 1.968 | 2.711 |
|  | 3 | -.518^*^ | .049 | .000 | -.688 | -.347 |
|  | 5 | -.063 | .053 | 1.000 | -.249 | .124 |
| 5 | 1 | -.445^*^ | .044 | .000 | -.600 | -.290 |
|  | 2 | 2.402^*^ | .103 | .000 | 2.042 | 2.762 |
|  | 3 | -.455^*^ | .046 | .000 | -.616 | -.294 |
|  | 4 | .063 | .053 | 1.000 | -.124 | .249 |

| Based on estimated marginal means^a^ |
| --- |
| *. The mean difference is significant at the .05 level. |
| a. Strain = E. coli |
| c. Adjustment for multiple comparisons: Bonferroni. |

| **Multivariate Tests^a^** | | | | | | |
| --- | --- | --- | --- | --- | --- | --- |
|  | Value | F | Hypothesis df | Error df | Sig. | Partial Eta Squared |
| Pillai's trace | .993 | 305.268^b^ | 4.000 | 8.000 | .000 | .993 |
| Wilks' lambda | .007 | 305.268^b^ | 4.000 | 8.000 | .000 | .993 |
| Hotelling's trace | 152.634 | 305.268^b^ | 4.000 | 8.000 | .000 | .993 |
| Roy's largest root | 152.634 | 305.268^b^ | 4.000 | 8.000 | .000 | .993 |

| Each F tests the multivariate effect of Extraction_method. These tests are based on the linearly independent pairwise comparisons among the estimated marginal means.^a^ |
| --- |
| a. Strain = E. coli |
| b. Exact statistic |

**Profile Plots**


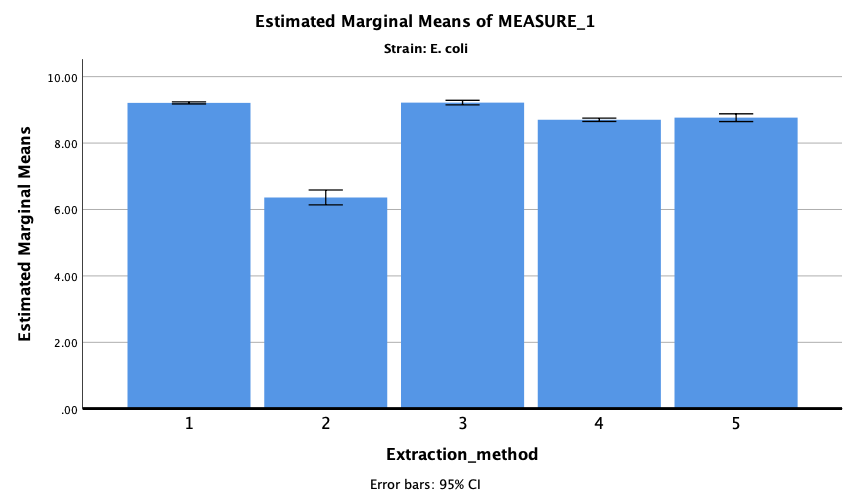


**Strain = E. faecalis**

| a. Strain = E. faecalis |
| --- |

| **Descriptive Statistics^a^** | | | |
| --- | --- | --- | --- |
|  | Mean | Std. Deviation | N |
| PheChl | 9.0517 | .37769 | 12 |
| Promega | 6.7208 | .41004 | 12 |
| Qiagen | 8.8450 | .18303 | 12 |
| C2mimOAc | 6.6308 | .12530 | 12 |
| ChoHex | 7.9000 | .10880 | 12 |

| a. Strain = E. faecalis |
| --- |

| **Multivariate Tests^a,b^** | | | | | | | |
| --- | --- | --- | --- | --- | --- | --- | --- |
| Effect | | Value | F | Hypothesis df | Error df | Sig. | Partial Eta Squared |
| Extraction_method | Pillai's Trace | .995 | 364.781^c^ | 4.000 | 8.000 | .000 | .995 |
|  | Wilks' Lambda | .005 | 364.781^c^ | 4.000 | 8.000 | .000 | .995 |
|  | Hotelling's Trace | 182.390 | 364.781^c^ | 4.000 | 8.000 | .000 | .995 |
|  | Roy's Largest Root | 182.390 | 364.781^c^ | 4.000 | 8.000 | .000 | .995 |

| a. Strain = E. faecalis |
| --- |
| b. Design: Intercept  Within Subjects Design: Extraction_method |
| c. Exact statistic |

| **Mauchly's Test of Sphericity^a,b^** | | | | | | | |
| --- | --- | --- | --- | --- | --- | --- | --- |
| Measure: MEASURE_1 | | | | | | | |
| Within Subjects Effect | Mauchly's W | Approx. Chi-Square | df | Sig. | Epsilon^c^ | |  |
|  |  |  |  |  | Greenhouse-Geisser | Huynh-Feldt |  |
| Extraction_method | .038 | 30.908 | 9 | .000 | .422 | .491 |  |

| **Tests of Within-Subjects Effects^a^** | | | | | | | |
| --- | --- | --- | --- | --- | --- | --- | --- |
| Measure: MEASURE_1 | | | | | | | |
| Source | | Type III Sum of Squares | df | Mean Square | F | Sig. |  |
| Extraction_method | Sphericity Assumed | 62.350 | 4 | 15.588 | 176.898 | .000 |  |
|  | Greenhouse-Geisser | 62.350 | 1.689 | 36.913 | 176.898 | .000 |  |
|  | Huynh-Feldt | 62.350 | 1.962 | 31.777 | 176.898 | .000 |  |
|  | Lower-bound | 62.350 | 1.000 | 62.350 | 176.898 | .000 |  |
| Error(Extraction_method) | Sphericity Assumed | 3.877 | 44 | .088 |  |  |  |
|  | Greenhouse-Geisser | 3.877 | 18.580 | .209 |  |  |  |
|  | Huynh-Feldt | 3.877 | 21.583 | .180 |  |  |  |
|  | Lower-bound | 3.877 | 11.000 | .352 |  |  |  |

| **Tests of Within-Subjects Contrasts^a^** | | | | | | | |
| --- | --- | --- | --- | --- | --- | --- | --- |
| Measure: MEASURE_1 | | | | | | | |
| Source | Extraction_method | Type III Sum of Squares | df | Mean Square | F | Sig. |  |
| Extraction_method | Linear | 6.874 | 1 | 6.874 | 141.931 | .000 |  |
|  | Quadratic | 7.019 | 1 | 7.019 | 56.486 | .000 |  |
|  | Cubic | 1.133 | 1 | 1.133 | 8.623 | .014 |  |
|  | Order 4 | 47.324 | 1 | 47.324 | 978.165 | .000 |  |
| Error(Extraction_method) | Linear | .533 | 11 | .048 |  |  |  |
|  | Quadratic | 1.367 | 11 | .124 |  |  |  |
|  | Cubic | 1.445 | 11 | .131 |  |  |  |
|  | Order 4 | .532 | 11 | .048 |  |  |  |

| **Tests of Between-Subjects Effects^a^** | | | | | | |
| --- | --- | --- | --- | --- | --- | --- |
| Measure: MEASURE_1 | | | | | | |
| Transformed Variable: Average | | | | | | |
| Source | Type III Sum of Squares | df | Mean Square | F | Sig. | Partial Eta Squared |
| Intercept | 3678.221 | 1 | 3678.221 | 189996.692 | .000 | 1.000 |
| Error | .213 | 11 | .019 |  |  |  |

| a. Strain = E. faecalis |
| --- |

**Estimated Marginal Means**

**Extraction_method**

| **Estimates^a^** | | | | |
| --- | --- | --- | --- | --- |
| Measure: MEASURE_1 | | | | |
| Extraction_method | Mean | Std. Error | 95% Confidence Interval | |
|  |  |  | Lower Bound | Upper Bound |
| 1 | 9.052 | .109 | 8.812 | 9.292 |
| 2 | 6.721 | .118 | 6.460 | 6.981 |
| 3 | 8.845 | .053 | 8.729 | 8.961 |
| 4 | 6.631 | .036 | 6.551 | 6.710 |
| 5 | 7.900 | .031 | 7.831 | 7.969 |
| a. Strain = E. faecalis | | | | |

| **Pairwise Comparisons^a^** | | | | | | |
| --- | --- | --- | --- | --- | --- | --- |
| Measure: MEASURE_1 | | | | | | |
| (I) Extraction_method | (J) Extraction_method | Mean Difference (I-J) | Std. Error | Sig.^c^ | 95% Confidence Interval for Difference^c^ | |
|  |  |  |  |  | Lower Bound | Upper Bound |
| 1 | 2 | 2.331^*^ | .206 | .000 | 1.611 | 3.050 |
|  | 3 | .207 | .140 | 1.000 | -.281 | .695 |
|  | 4 | 2.421^*^ | .113 | .000 | 2.024 | 2.818 |
|  | 5 | 1.152^*^ | .106 | .000 | .780 | 1.523 |
| 2 | 1 | -2.331^*^ | .206 | .000 | -3.050 | -1.611 |
|  | 3 | -2.124^*^ | .114 | .000 | -2.523 | -1.726 |
|  | 4 | .090 | .137 | 1.000 | -.388 | .568 |
|  | 5 | -1.179^*^ | .137 | .000 | -1.660 | -.699 |
| 3 | 1 | -.207 | .140 | 1.000 | -.695 | .281 |
|  | 2 | 2.124^*^ | .114 | .000 | 1.726 | 2.523 |
|  | 4 | 2.214^*^ | .071 | .000 | 1.966 | 2.463 |
|  | 5 | .945^*^ | .064 | .000 | .721 | 1.169 |
| 4 | 1 | -2.421^*^ | .113 | .000 | -2.818 | -2.024 |
|  | 2 | -.090 | .137 | 1.000 | -.568 | .388 |
|  | 3 | -2.214^*^ | .071 | .000 | -2.463 | -1.966 |
|  | 5 | -1.269^*^ | .034 | .000 | -1.388 | -1.151 |
| 5 | 1 | -1.152^*^ | .106 | .000 | -1.523 | -.780 |
|  | 2 | 1.179^*^ | .137 | .000 | .699 | 1.660 |
|  | 3 | -.945^*^ | .064 | .000 | -1.169 | -.721 |
|  | 4 | 1.269^*^ | .034 | .000 | 1.151 | 1.388 |

| Based on estimated marginal means^a^ |
| --- |
| *. The mean difference is significant at the .05 level. |
| a. Strain = E. faecalis |
| c. Adjustment for multiple comparisons: Bonferroni. |

| **Multivariate Tests^a^** | | | | | | |
| --- | --- | --- | --- | --- | --- | --- |
|  | Value | F | Hypothesis df | Error df | Sig. | Partial Eta Squared |
| Pillai's trace | .995 | 364.781^b^ | 4.000 | 8.000 | .000 | .995 |
| Wilks' lambda | .005 | 364.781^b^ | 4.000 | 8.000 | .000 | .995 |
| Hotelling's trace | 182.390 | 364.781^b^ | 4.000 | 8.000 | .000 | .995 |
| Roy's largest root | 182.390 | 364.781^b^ | 4.000 | 8.000 | .000 | .995 |

| Each F tests the multivariate effect of Extraction_method. These tests are based on the linearly independent pairwise comparisons among the estimated marginal means.^a^ |
| --- |
| a. Strain = E. faecalis |
| b. Exact statistic |

**Profile Plots**


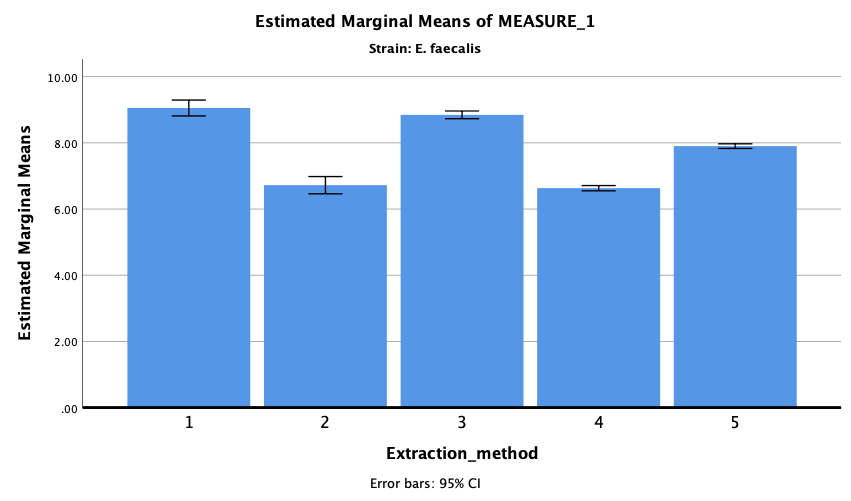


**Strain = L. pneumophila**

| **Between-Subjects Factors^a^** |
| --- |

| a. Strain = L. pneumophila |
| --- |

| **Descriptive Statistics^a^** | | | |
| --- | --- | --- | --- |
|  | Mean | Std. Deviation | N |
| PheChl | 9.2908 | .08723 | 12 |
| Promega | 7.9408 | .19000 | 12 |
| Qiagen | 9.3467 | .07365 | 12 |
| C2mimOAc | 8.7317 | .60376 | 12 |
| ChoHex | 8.8583 | .07457 | 12 |

| a. Strain = L. pneumophila |
| --- |

| **Multivariate Tests^a,b^** | | | | | | | |
| --- | --- | --- | --- | --- | --- | --- | --- |
| Effect | | Value | F | Hypothesis df | Error df | Sig. | Partial Eta Squared |
| Extraction_method | Pillai's Trace | .990 | 202.095^c^ | 4.000 | 8.000 | .000 | .990 |
|  | Wilks' Lambda | .010 | 202.095^c^ | 4.000 | 8.000 | .000 | .990 |
|  | Hotelling's Trace | 101.048 | 202.095^c^ | 4.000 | 8.000 | .000 | .990 |
|  | Roy's Largest Root | 101.048 | 202.095^c^ | 4.000 | 8.000 | .000 | .990 |

| a. Strain = L. pneumophila |
| --- |
| b. Design: Intercept  Within Subjects Design: Extraction_method |
| c. Exact statistic |

| **Mauchly's Test of Sphericity^a,b^** | | | | | | | |
| --- | --- | --- | --- | --- | --- | --- | --- |
| Measure: MEASURE_1 | | | | | | | |
| Within Subjects Effect | Mauchly's W | Approx. Chi-Square | df | Sig. | Epsilon^c^ | |  |
|  |  |  |  |  | Greenhouse-Geisser | Huynh-Feldt |  |
| Extraction_method | .001 | 61.732 | 9 | .000 | .291 | .304 |  |

| **Tests of Within-Subjects Effects^a^** | | | | | | | |
| --- | --- | --- | --- | --- | --- | --- | --- |
| Measure: MEASURE_1 | | | | | | | |
| Source | | Type III Sum of Squares | df | Mean Square | F | Sig. |  |
| Extraction_method | Sphericity Assumed | 15.364 | 4 | 3.841 | 48.582 | .000 |  |
|  | Greenhouse-Geisser | 15.364 | 1.162 | 13.219 | 48.582 | .000 |  |
|  | Huynh-Feldt | 15.364 | 1.214 | 12.651 | 48.582 | .000 |  |
|  | Lower-bound | 15.364 | 1.000 | 15.364 | 48.582 | .000 |  |
| Error(Extraction_method) | Sphericity Assumed | 3.479 | 44 | .079 |  |  |  |
|  | Greenhouse-Geisser | 3.479 | 12.785 | .272 |  |  |  |
|  | Huynh-Feldt | 3.479 | 13.359 | .260 |  |  |  |
|  | Lower-bound | 3.479 | 11.000 | .316 |  |  |  |

| **Tests of Within-Subjects Contrasts^a^** | | | | | | | |
| --- | --- | --- | --- | --- | --- | --- | --- |
| Measure: MEASURE_1 | | | | | | | |
| Source | Extraction_method | Type III Sum of Squares | df | Mean Square | F | Sig. |  |
| Extraction_method | Linear | .007 | 1 | .007 | .215 | .652 |  |
|  | Quadratic | .745 | 1 | .745 | 10.531 | .008 |  |
|  | Cubic | 4.868 | 1 | 4.868 | 47.028 | .000 |  |
|  | Order 4 | 9.744 | 1 | 9.744 | 87.628 | .000 |  |
| Error(Extraction_method) | Linear | .338 | 11 | .031 |  |  |  |
|  | Quadratic | .779 | 11 | .071 |  |  |  |
|  | Cubic | 1.139 | 11 | .104 |  |  |  |
|  | Order 4 | 1.223 | 11 | .111 |  |  |  |

| **Tests of Between-Subjects Effects^a^** | | | | | | |
| --- | --- | --- | --- | --- | --- | --- |
| Measure: MEASURE_1 | | | | | | |
| Transformed Variable: Average | | | | | | |
| Source | Type III Sum of Squares | df | Mean Square | F | Sig. | Partial Eta Squared |
| Intercept | 4682.020 | 1 | 4682.020 | 45471.221 | .000 | 1.000 |
| Error | 1.133 | 11 | .103 |  |  |  |

| a. Strain = L. pneumophila |
| --- |

**Estimated Marginal Means**

**Extraction_method**

| **Estimates^a^** | | | | |
| --- | --- | --- | --- | --- |
| Measure: MEASURE_1 | | | | |
| Extraction_method | Mean | Std. Error | 95% Confidence Interval | |
|  |  |  | Lower Bound | Upper Bound |
| 1 | 9.291 | .025 | 9.235 | 9.346 |
| 2 | 7.941 | .055 | 7.820 | 8.062 |
| 3 | 9.347 | .021 | 9.300 | 9.393 |
| 4 | 8.732 | .174 | 8.348 | 9.115 |
| 5 | 8.858 | .022 | 8.811 | 8.906 |

| a. Strain = L. pneumophila |
| --- |

| **Pairwise Comparisons^a^** | | | | | | |
| --- | --- | --- | --- | --- | --- | --- |
| Measure: MEASURE_1 | | | | | | |
| (I) Extraction_method | (J) Extraction_method | Mean Difference (I-J) | Std. Error | Sig.^c^ | 95% Confidence Interval for Difference^c^ | |
|  |  |  |  |  | Lower Bound | Upper Bound |
| 1 | 2 | 1.350^*^ | .061 | .000 | 1.136 | 1.564 |
|  | 3 | -.056 | .034 | 1.000 | -.175 | .063 |
|  | 4 | .559 | .188 | .128 | -.100 | 1.218 |
|  | 5 | .432^*^ | .024 | .000 | .349 | .516 |
| 2 | 1 | -1.350^*^ | .061 | .000 | -1.564 | -1.136 |
|  | 3 | -1.406^*^ | .051 | .000 | -1.585 | -1.226 |
|  | 4 | -.791^*^ | .148 | .002 | -1.307 | -.275 |
|  | 5 | -.917^*^ | .060 | .000 | -1.128 | -.707 |
| 3 | 1 | .056 | .034 | 1.000 | -.063 | .175 |
|  | 2 | 1.406^*^ | .051 | .000 | 1.226 | 1.585 |
|  | 4 | .615^*^ | .163 | .031 | .044 | 1.186 |
|  | 5 | .488^*^ | .037 | .000 | .358 | .619 |
| 4 | 1 | -.559 | .188 | .128 | -1.218 | .100 |
|  | 2 | .791^*^ | .148 | .002 | .275 | 1.307 |
|  | 3 | -.615^*^ | .163 | .031 | -1.186 | -.044 |
|  | 5 | -.127 | .186 | 1.000 | -.777 | .524 |
| 5 | 1 | -.432^*^ | .024 | .000 | -.516 | -.349 |
|  | 2 | .917^*^ | .060 | .000 | .707 | 1.128 |
|  | 3 | -.488^*^ | .037 | .000 | -.619 | -.358 |
|  | 4 | .127 | .186 | 1.000 | -.524 | .777 |

| Based on estimated marginal means^a^ |
| --- |
| *. The mean difference is significant at the .05 level. |
| a. Strain = L. pneumophila |
| c. Adjustment for multiple comparisons: Bonferroni. |

| **Multivariate Tests^a^** | | | | | | |
| --- | --- | --- | --- | --- | --- | --- |
|  | Value | F | Hypothesis df | Error df | Sig. | Partial Eta Squared |
| Pillai's trace | .990 | 202.095^b^ | 4.000 | 8.000 | .000 | .990 |
| Wilks' lambda | .010 | 202.095^b^ | 4.000 | 8.000 | .000 | .990 |
| Hotelling's trace | 101.048 | 202.095^b^ | 4.000 | 8.000 | .000 | .990 |
| Roy's largest root | 101.048 | 202.095^b^ | 4.000 | 8.000 | .000 | .990 |

| Each F tests the multivariate effect of Extraction_method. These tests are based on the linearly independent pairwise comparisons among the estimated marginal means.^a^ |
| --- |
| a. Strain = L. pneumophila |
| b. Exact statistic |

**Profile Plots**


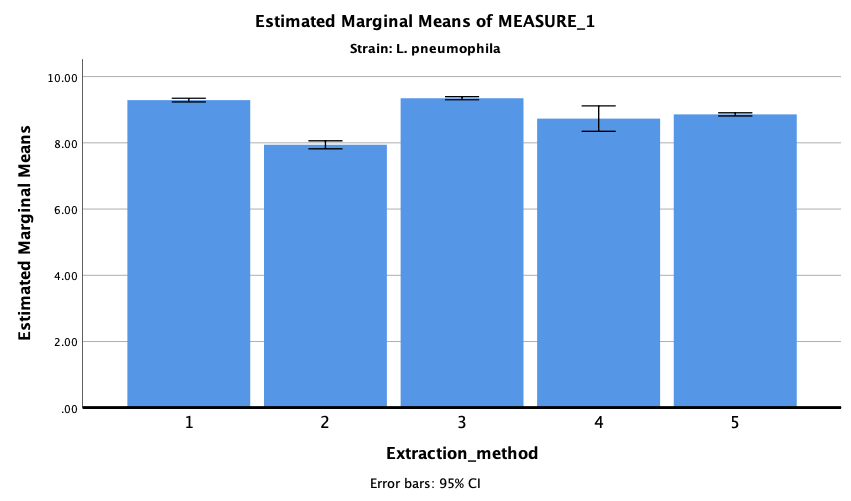


**Strain = P. aeruginosa**

| a. Strain = P. aeruginosa |
| --- |

| **Descriptive Statistics^a^** | | | |
| --- | --- | --- | --- |
|  | Mean | Std. Deviation | N |
| PheChl | 8.9050 | .06990 | 8 |
| Promega | 6.5238 | .49065 | 8 |
| Qiagen | 8.7575 | .09114 | 8 |
| C2mimOAc | 8.2600 | .24107 | 8 |
| ChoHex | 8.6725 | .13573 | 8 |

| a. Strain = P. aeruginosa |
| --- |

| **Multivariate Tests^a,b^** | | | | | | | |
| --- | --- | --- | --- | --- | --- | --- | --- |
| Effect | | Value | F | Hypothesis df | Error df | Sig. | Partial Eta Squared |
| Extraction_method | Pillai's Trace | .996 | 240.337^c^ | 4.000 | 4.000 | .000 | .996 |
|  | Wilks' Lambda | .004 | 240.337^c^ | 4.000 | 4.000 | .000 | .996 |
|  | Hotelling's Trace | 240.337 | 240.337^c^ | 4.000 | 4.000 | .000 | .996 |
|  | Roy's Largest Root | 240.337 | 240.337^c^ | 4.000 | 4.000 | .000 | .996 |

| a. Strain = P. aeruginosa |
| --- |
| b. Design: Intercept  Within Subjects Design: Extraction_method |
| c. Exact statistic |

| **Mauchly's Test of Sphericity^a,b^** | | | | | | | |
| --- | --- | --- | --- | --- | --- | --- | --- |
| Measure: MEASURE_1 | | | | | | | |
| Within Subjects Effect | Mauchly's W | Approx. Chi-Square | df | Sig. | Epsilon^c^ | |  |
|  |  |  |  |  | Greenhouse-Geisser | Huynh-Feldt |  |
| Extraction_method | .001 | 38.057 | 9 | .000 | .318 | .357 |  |

| **Tests of Within-Subjects Effects^a^** | | | | | | | |
| --- | --- | --- | --- | --- | --- | --- | --- |
| Measure: MEASURE_1 | | | | | | | |
| Source | | Type III Sum of Squares | df | Mean Square | F | Sig. |  |
| Extraction_method | Sphericity Assumed | 30.733 | 4 | 7.683 | 121.862 | .000 |  |
|  | Greenhouse-Geisser | 30.733 | 1.272 | 24.164 | 121.862 | .000 |  |
|  | Huynh-Feldt | 30.733 | 1.427 | 21.535 | 121.862 | .000 |  |
|  | Lower-bound | 30.733 | 1.000 | 30.733 | 121.862 | .000 |  |
| Error(Extraction_method) | Sphericity Assumed | 1.765 | 28 | .063 |  |  |  |
|  | Greenhouse-Geisser | 1.765 | 8.903 | .198 |  |  |  |
|  | Huynh-Feldt | 1.765 | 9.990 | .177 |  |  |  |
|  | Lower-bound | 1.765 | 7.000 | .252 |  |  |  |

| **Tests of Within-Subjects Contrasts^a^** | | | | | | | |
| --- | --- | --- | --- | --- | --- | --- | --- |
| Measure: MEASURE_1 | | | | | | | |
| Source | Extraction_method | Type III Sum of Squares | df | Mean Square | F | Sig. |  |
| Extraction_method | Linear | 1.293 | 1 | 1.293 | 73.215 | .000 |  |
|  | Quadratic | 4.662 | 1 | 4.662 | 50.298 | .000 |  |
|  | Cubic | 10.982 | 1 | 10.982 | 230.596 | .000 |  |
|  | Order 4 | 13.797 | 1 | 13.797 | 146.414 | .000 |  |
| Error(Extraction_method) | Linear | .124 | 7 | .018 |  |  |  |
|  | Quadratic | .649 | 7 | .093 |  |  |  |
|  | Cubic | .333 | 7 | .048 |  |  |  |
|  | Order 4 | .660 | 7 | .094 |  |  |  |

| **Tests of Between-Subjects Effects^a^** | | | | | | |
| --- | --- | --- | --- | --- | --- | --- |
| Measure: MEASURE_1 | | | | | | |
| Transformed Variable: Average | | | | | | |
| Source | Type III Sum of Squares | df | Mean Square | F | Sig. | Partial Eta Squared |
| Intercept | 2705.203 | 1 | 2705.203 | 34561.972 | .000 | 1.000 |
| Error | .548 | 7 | .078 |  |  |  |

| a. Strain = P. aeruginosa |
| --- |

**Estimated Marginal Means**

**Extraction_method**

| **Estimates^a^** | | | | |
| --- | --- | --- | --- | --- |
| Measure: MEASURE_1 | | | | |
| Extraction_method | Mean | Std. Error | 95% Confidence Interval | |
|  |  |  | Lower Bound | Upper Bound |
| 1 | 8.905 | .025 | 8.847 | 8.963 |
| 2 | 6.524 | .173 | 6.114 | 6.934 |
| 3 | 8.758 | .032 | 8.681 | 8.834 |
| 4 | 8.260 | .085 | 8.058 | 8.462 |
| 5 | 8.673 | .048 | 8.559 | 8.786 |

| a. Strain = P. aeruginosa |
| --- |

| **Pairwise Comparisons^a^** | | | | | | |
| --- | --- | --- | --- | --- | --- | --- |
| Measure: MEASURE_1 | | | | | | |
| (I) Extraction_method | (J) Extraction_method | Mean Difference (I-J) | Std. Error | Sig.^c^ | 95% Confidence Interval for Difference^c^ | |
|  |  |  |  |  | Lower Bound | Upper Bound |
| 1 | 2 | 2.381^*^ | .191 | .000 | 1.612 | 3.150 |
|  | 3 | .147 | .050 | .223 | -.056 | .351 |
|  | 4 | .645^*^ | .096 | .003 | .257 | 1.033 |
|  | 5 | .232^*^ | .047 | .016 | .044 | .421 |
| 2 | 1 | -2.381^*^ | .191 | .000 | -3.150 | -1.612 |
|  | 3 | -2.234^*^ | .148 | .000 | -2.830 | -1.637 |
|  | 4 | -1.736^*^ | .119 | .000 | -2.215 | -1.257 |
|  | 5 | -2.149^*^ | .209 | .000 | -2.992 | -1.305 |
| 3 | 1 | -.147 | .050 | .223 | -.351 | .056 |
|  | 2 | 2.234^*^ | .148 | .000 | 1.637 | 2.830 |
|  | 4 | .498^*^ | .077 | .003 | .188 | .807 |
|  | 5 | .085 | .066 | 1.000 | -.181 | .351 |
| 4 | 1 | -.645^*^ | .096 | .003 | -1.033 | -.257 |
|  | 2 | 1.736^*^ | .119 | .000 | 1.257 | 2.215 |
|  | 3 | -.498^*^ | .077 | .003 | -.807 | -.188 |
|  | 5 | -.412 | .131 | .159 | -.938 | .113 |
| 5 | 1 | -.232^*^ | .047 | .016 | -.421 | -.044 |
|  | 2 | 2.149^*^ | .209 | .000 | 1.305 | 2.992 |
|  | 3 | -.085 | .066 | 1.000 | -.351 | .181 |
|  | 4 | .412 | .131 | .159 | -.113 | .938 |

| Based on estimated marginal means^a^ |
| --- |
| *. The mean difference is significant at the .05 level. |
| a. Strain = P. aeruginosa |
| c. Adjustment for multiple comparisons: Bonferroni. |

| **Multivariate Tests^a^** | | | | | | |
| --- | --- | --- | --- | --- | --- | --- |
|  | Value | F | Hypothesis df | Error df | Sig. | Partial Eta Squared |
| Pillai's trace | .996 | 240.337^b^ | 4.000 | 4.000 | .000 | .996 |
| Wilks' lambda | .004 | 240.337^b^ | 4.000 | 4.000 | .000 | .996 |
| Hotelling's trace | 240.337 | 240.337^b^ | 4.000 | 4.000 | .000 | .996 |
| Roy's largest root | 240.337 | 240.337^b^ | 4.000 | 4.000 | .000 | .996 |

| Each F tests the multivariate effect of Extraction_method. These tests are based on the linearly independent pairwise comparisons among the estimated marginal means.^a^ |
| --- |
| a. Strain = P. aeruginosa |
| b. Exact statistic |

**Profile Plots**


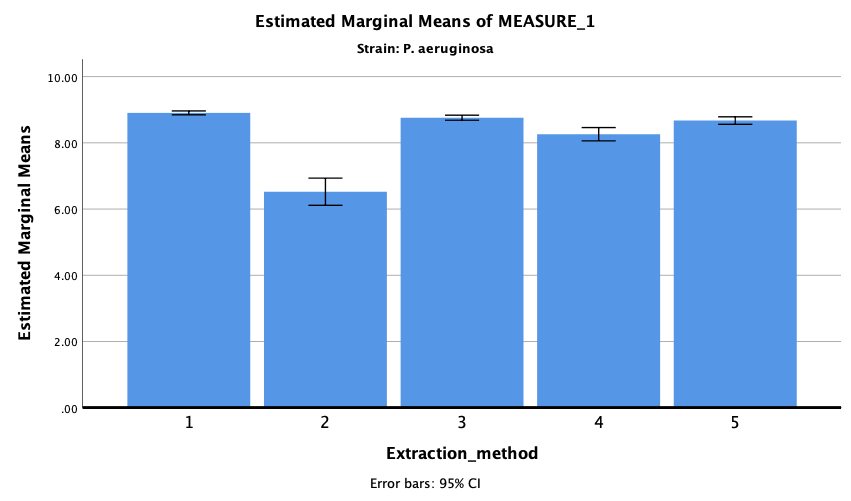


**Strain = S. aureus**

| a. Strain = S. aureus |
| --- |

| **Descriptive Statistics^a^** | | | |
| --- | --- | --- | --- |
|  | Mean | Std. Deviation | N |
| PheChl | 8.2067 | .22342 | 12 |
| Promega | 5.9692 | .44525 | 12 |
| Qiagen | 8.5367 | .19635 | 12 |
| C2mimOAc | 7.6200 | .22579 | 12 |
| ChoHex | 8.2967 | .06919 | 12 |

| a. Strain = S. aureus |
| --- |

| **Multivariate Tests^a,b^** | | | | | | | |
| --- | --- | --- | --- | --- | --- | --- | --- |
| Effect | | Value | F | Hypothesis df | Error df | Sig. | Partial Eta Squared |
| Extraction_method | Pillai's Trace | .994 | 350.526^c^ | 4.000 | 8.000 | .000 | .994 |
|  | Wilks' Lambda | .006 | 350.526^c^ | 4.000 | 8.000 | .000 | .994 |
|  | Hotelling's Trace | 175.263 | 350.526^c^ | 4.000 | 8.000 | .000 | .994 |
|  | Roy's Largest Root | 175.263 | 350.526^c^ | 4.000 | 8.000 | .000 | .994 |

| a. Strain = S. aureus |
| --- |
| b. Design: Intercept  Within Subjects Design: Extraction_method |
| c. Exact statistic |

| **Mauchly's Test of Sphericity^a,b^** | | | | | | | |
| --- | --- | --- | --- | --- | --- | --- | --- |
| Measure: MEASURE_1 | | | | | | | |
| Within Subjects Effect | Mauchly's W | Approx. Chi-Square | df | Sig. | Epsilon^c^ | |  |
|  |  |  |  |  | Greenhouse-Geisser | Huynh-Feldt |  |
| Extraction_method | .009 | 44.223 | 9 | .000 | .448 | .530 |  |

| **Tests of Within-Subjects Effects^a^** | | | | | | | |
| --- | --- | --- | --- | --- | --- | --- | --- |
| Measure: MEASURE_1 | | | | | | | |
| Source | | Type III Sum of Squares | df | Mean Square | F | Sig. |  |
| Extraction_method | Sphericity Assumed | 51.739 | 4 | 12.935 | 195.497 | .000 |  |
|  | Greenhouse-Geisser | 51.739 | 1.794 | 28.845 | 195.497 | .000 |  |
|  | Huynh-Feldt | 51.739 | 2.121 | 24.397 | 195.497 | .000 |  |
|  | Lower-bound | 51.739 | 1.000 | 51.739 | 195.497 | .000 |  |
| Error(Extraction_method) | Sphericity Assumed | 2.911 | 44 | .066 |  |  |  |
|  | Greenhouse-Geisser | 2.911 | 19.731 | .148 |  |  |  |
|  | Huynh-Feldt | 2.911 | 23.328 | .125 |  |  |  |
|  | Lower-bound | 2.911 | 11.000 | .265 |  |  |  |

| **Tests of Within-Subjects Contrasts^a^** | | | | | | | |
| --- | --- | --- | --- | --- | --- | --- | --- |
| Measure: MEASURE_1 | | | | | | | |
| Source | Extraction_method | Type III Sum of Squares | df | Mean Square | F | Sig. |  |
| Extraction_method | Linear | 4.022 | 1 | 4.022 | 269.054 | .000 |  |
|  | Quadratic | 4.710 | 1 | 4.710 | 54.066 | .000 |  |
|  | Cubic | 12.378 | 1 | 12.378 | 110.319 | .000 |  |
|  | Order 4 | 30.629 | 1 | 30.629 | 607.872 | .000 |  |
| Error(Extraction_method) | Linear | .164 | 11 | .015 |  |  |  |
|  | Quadratic | .958 | 11 | .087 |  |  |  |
|  | Cubic | 1.234 | 11 | .112 |  |  |  |
|  | Order 4 | .554 | 11 | .050 |  |  |  |

| **Tests of Between-Subjects Effects^a^** | | | | | | |
| --- | --- | --- | --- | --- | --- | --- |
| Measure: MEASURE_1 | | | | | | |
| Transformed Variable: Average | | | | | | |
| Source | Type III Sum of Squares | df | Mean Square | F | Sig. | Partial Eta Squared |
| Intercept | 3581.310 | 1 | 3581.310 | 46016.221 | .000 | 1.000 |
| Error | .856 | 11 | .078 |  |  |  |

| a. Strain = S. aureus |
| --- |

**Estimated Marginal Means**

**Extraction_method**

| **Estimates^a^** | | | | |
| --- | --- | --- | --- | --- |
| Measure: MEASURE_1 | | | | |
| Extraction_method | Mean | Std. Error | 95% Confidence Interval | |
|  |  |  | Lower Bound | Upper Bound |
| 1 | 8.207 | .064 | 8.065 | 8.349 |
| 2 | 5.969 | .129 | 5.686 | 6.252 |
| 3 | 8.537 | .057 | 8.412 | 8.661 |
| 4 | 7.620 | .065 | 7.477 | 7.763 |
| 5 | 8.297 | .020 | 8.253 | 8.341 |

| a. Strain = S. aureus |
| --- |

| **Pairwise Comparisons^a^** | | | | | | |
| --- | --- | --- | --- | --- | --- | --- |
| Measure: MEASURE_1 | | | | | | |
| (I) Extraction_method | (J) Extraction_method | Mean Difference (I-J) | Std. Error | Sig.^c^ | 95% Confidence Interval for Difference^c^ | |
|  |  |  |  |  | Lower Bound | Upper Bound |
| 1 | 2 | 2.237^*^ | .162 | .000 | 1.669 | 2.806 |
|  | 3 | -.330 | .111 | .126 | -.718 | .058 |
|  | 4 | .587^*^ | .076 | .000 | .320 | .854 |
|  | 5 | -.090 | .056 | 1.000 | -.285 | .105 |
| 2 | 1 | -2.237^*^ | .162 | .000 | -2.806 | -1.669 |
|  | 3 | -2.567^*^ | .094 | .000 | -2.897 | -2.238 |
|  | 4 | -1.651^*^ | .134 | .000 | -2.121 | -1.181 |
|  | 5 | -2.327^*^ | .129 | .000 | -2.778 | -1.877 |
| 3 | 1 | .330 | .111 | .126 | -.058 | .718 |
|  | 2 | 2.567^*^ | .094 | .000 | 2.238 | 2.897 |
|  | 4 | .917^*^ | .103 | .000 | .558 | 1.275 |
|  | 5 | .240^*^ | .059 | .018 | .035 | .445 |
| 4 | 1 | -.587^*^ | .076 | .000 | -.854 | -.320 |
|  | 2 | 1.651^*^ | .134 | .000 | 1.181 | 2.121 |
|  | 3 | -.917^*^ | .103 | .000 | -1.275 | -.558 |
|  | 5 | -.677^*^ | .072 | .000 | -.927 | -.427 |
| 5 | 1 | .090 | .056 | 1.000 | -.105 | .285 |
|  | 2 | 2.327^*^ | .129 | .000 | 1.877 | 2.778 |
|  | 3 | -.240^*^ | .059 | .018 | -.445 | -.035 |
|  | 4 | .677^*^ | .072 | .000 | .427 | .927 |

| Based on estimated marginal means^a^ |
| --- |
| *. The mean difference is significant at the .05 level. |
| a. Strain = S. aureus |
| c. Adjustment for multiple comparisons: Bonferroni. |

| **Multivariate Tests^a^** | | | | | | |
| --- | --- | --- | --- | --- | --- | --- |
|  | Value | F | Hypothesis df | Error df | Sig. | Partial Eta Squared |
| Pillai's trace | .994 | 350.526^b^ | 4.000 | 8.000 | .000 | .994 |
| Wilks' lambda | .006 | 350.526^b^ | 4.000 | 8.000 | .000 | .994 |
| Hotelling's trace | 175.263 | 350.526^b^ | 4.000 | 8.000 | .000 | .994 |
| Roy's largest root | 175.263 | 350.526^b^ | 4.000 | 8.000 | .000 | .994 |

| Each F tests the multivariate effect of Extraction_method. These tests are based on the linearly independent pairwise comparisons among the estimated marginal means.^a^ |
| --- |
| a. Strain = S. aureus |
| b. Exact statistic |

**Profile Plots**


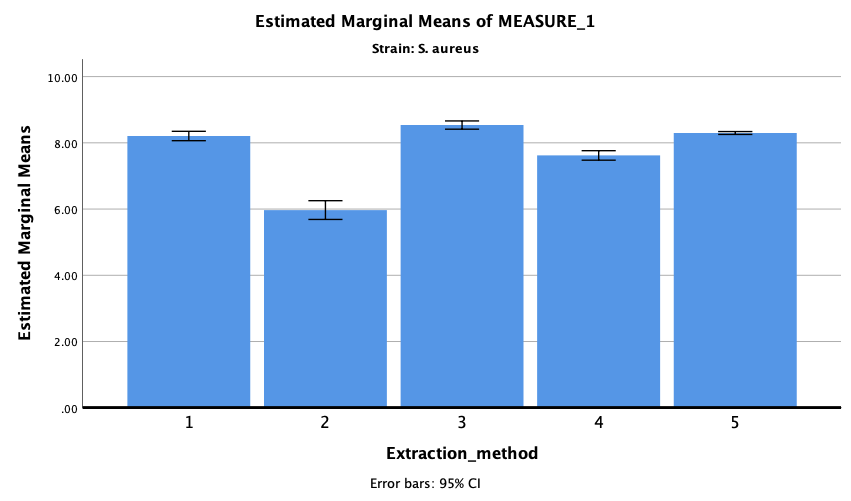


**Strain = V. cholerae**

| a. Strain = V. cholerae |
| --- |

| **Descriptive Statistics^a^** | | | |
| --- | --- | --- | --- |
|  | Mean | Std. Deviation | N |
| PheChl | 9.2483 | .04687 | 12 |
| Promega | 7.2292 | .66918 | 12 |
| Qiagen | 9.2717 | .04896 | 12 |
| C2mimOAc | 8.5683 | .18780 | 12 |
| ChoHex | 9.0400 | .08676 | 12 |

| a. Strain = V. cholerae |
| --- |

| **Multivariate Tests^a,b^** | | | | | | | |
| --- | --- | --- | --- | --- | --- | --- | --- |
| Effect | | Value | F | Hypothesis df | Error df | Sig. | Partial Eta Squared |
| Extraction_method | Pillai's Trace | .984 | 125.858^c^ | 4.000 | 8.000 | .000 | .984 |
|  | Wilks' Lambda | .016 | 125.858^c^ | 4.000 | 8.000 | .000 | .984 |
|  | Hotelling's Trace | 62.929 | 125.858^c^ | 4.000 | 8.000 | .000 | .984 |
|  | Roy's Largest Root | 62.929 | 125.858^c^ | 4.000 | 8.000 | .000 | .984 |

| a. Strain = V. cholerae |
| --- |
| b. Design: Intercept  Within Subjects Design: Extraction_method |
| c. Exact statistic |

| **Mauchly's Test of Sphericity^a,b^** | | | | | | | |
| --- | --- | --- | --- | --- | --- | --- | --- |
| Measure: MEASURE_1 | | | | | | | |
| Within Subjects Effect | Mauchly's W | Approx. Chi-Square | df | Sig. | Epsilon^c^ | |  |
|  |  |  |  |  | Greenhouse-Geisser | Huynh-Feldt |  |
| Extraction_method | .000 | 80.964 | 9 | .000 | .304 | .322 |  |

| **Tests of Within-Subjects Effects^a^** | | | | | | | |
| --- | --- | --- | --- | --- | --- | --- | --- |
| Measure: MEASURE_1 | | | | | | | |
| Source | | Type III Sum of Squares | df | Mean Square | F | Sig. |  |
| Extraction_method | Sphericity Assumed | 35.036 | 4 | 8.759 | 93.408 | .000 |  |
|  | Greenhouse-Geisser | 35.036 | 1.218 | 28.771 | 93.408 | .000 |  |
|  | Huynh-Feldt | 35.036 | 1.289 | 27.173 | 93.408 | .000 |  |
|  | Lower-bound | 35.036 | 1.000 | 35.036 | 93.408 | .000 |  |
| Error(Extraction_method) | Sphericity Assumed | 4.126 | 44 | .094 |  |  |  |
|  | Greenhouse-Geisser | 4.126 | 13.395 | .308 |  |  |  |
|  | Huynh-Feldt | 4.126 | 14.183 | .291 |  |  |  |
|  | Lower-bound | 4.126 | 11.000 | .375 |  |  |  |

| **Tests of Within-Subjects Contrasts^a^** | | | | | | | |
| --- | --- | --- | --- | --- | --- | --- | --- |
| Measure: MEASURE_1 | | | | | | | |
| Source | Extraction_method | Type III Sum of Squares | df | Mean Square | F | Sig. |  |
| Extraction_method | Linear | 1.021 | 1 | 1.021 | 20.273 | .001 |  |
|  | Quadratic | 4.285 | 1 | 4.285 | 94.098 | .000 |  |
|  | Cubic | 9.999 | 1 | 9.999 | 57.139 | .000 |  |
|  | Order 4 | 19.731 | 1 | 19.731 | 189.395 | .000 |  |
| Error(Extraction_method) | Linear | .554 | 11 | .050 |  |  |  |
|  | Quadratic | .501 | 11 | .046 |  |  |  |
|  | Cubic | 1.925 | 11 | .175 |  |  |  |
|  | Order 4 | 1.146 | 11 | .104 |  |  |  |

| **Tests of Between-Subjects Effects^a^** | | | | | | |
| --- | --- | --- | --- | --- | --- | --- |
| Measure: MEASURE_1 | | | | | | |
| Transformed Variable: Average | | | | | | |
| Source | Type III Sum of Squares | df | Mean Square | F | Sig. | Partial Eta Squared |
| Intercept | 4511.695 | 1 | 4511.695 | 37563.166 | .000 | 1.000 |
| Error | 1.321 | 11 | .120 |  |  |  |

| a. Strain = V. cholerae |
| --- |

**Estimated Marginal Means**

**Extraction_method**

| **Estimates^a^** | | | | |
| --- | --- | --- | --- | --- |
| Measure: MEASURE_1 | | | | |
| Extraction_method | Mean | Std. Error | 95% Confidence Interval | |
|  |  |  | Lower Bound | Upper Bound |
| 1 | 9.248 | .014 | 9.219 | 9.278 |
| 2 | 7.229 | .193 | 6.804 | 7.654 |
| 3 | 9.272 | .014 | 9.241 | 9.303 |
| 4 | 8.568 | .054 | 8.449 | 8.688 |
| 5 | 9.040 | .025 | 8.985 | 9.095 |

| a. Strain = V. cholerae |
| --- |

| **Pairwise Comparisons^a^** | | | | | | |
| --- | --- | --- | --- | --- | --- | --- |
| Measure: MEASURE_1 | | | | | | |
| (I) Extraction_method | (J) Extraction_method | Mean Difference (I-J) | Std. Error | Sig.^c^ | 95% Confidence Interval for Difference^c^ | |
|  |  |  |  |  | Lower Bound | Upper Bound |
| 1 | 2 | 2.019^*^ | .188 | .000 | 1.362 | 2.676 |
|  | 3 | -.023 | .011 | .515 | -.061 | .014 |
|  | 4 | .680^*^ | .058 | .000 | .479 | .881 |
|  | 5 | .208^*^ | .022 | .000 | .133 | .284 |
| 2 | 1 | -2.019^*^ | .188 | .000 | -2.676 | -1.362 |
|  | 3 | -2.042^*^ | .182 | .000 | -2.680 | -1.405 |
|  | 4 | -1.339^*^ | .193 | .000 | -2.013 | -.666 |
|  | 5 | -1.811^*^ | .195 | .000 | -2.493 | -1.128 |
| 3 | 1 | .023 | .011 | .515 | -.014 | .061 |
|  | 2 | 2.042^*^ | .182 | .000 | 1.405 | 2.680 |
|  | 4 | .703^*^ | .059 | .000 | .497 | .910 |
|  | 5 | .232^*^ | .024 | .000 | .147 | .317 |
| 4 | 1 | -.680^*^ | .058 | .000 | -.881 | -.479 |
|  | 2 | 1.339^*^ | .193 | .000 | .666 | 2.013 |
|  | 3 | -.703^*^ | .059 | .000 | -.910 | -.497 |
|  | 5 | -.472^*^ | .068 | .000 | -.709 | -.234 |
| 5 | 1 | -.208^*^ | .022 | .000 | -.284 | -.133 |
|  | 2 | 1.811^*^ | .195 | .000 | 1.128 | 2.493 |
|  | 3 | -.232^*^ | .024 | .000 | -.317 | -.147 |
|  | 4 | .472^*^ | .068 | .000 | .234 | .709 |

| Based on estimated marginal means^a^ |
| --- |
| *. The mean difference is significant at the .05 level. |
| a. Strain = V. cholerae |
| c. Adjustment for multiple comparisons: Bonferroni. |

| **Multivariate Tests^a^** | | | | | | |
| --- | --- | --- | --- | --- | --- | --- |
|  | Value | F | Hypothesis df | Error df | Sig. | Partial Eta Squared |
| Pillai's trace | .984 | 125.858^b^ | 4.000 | 8.000 | .000 | .984 |
| Wilks' lambda | .016 | 125.858^b^ | 4.000 | 8.000 | .000 | .984 |
| Hotelling's trace | 62.929 | 125.858^b^ | 4.000 | 8.000 | .000 | .984 |
| Roy's largest root | 62.929 | 125.858^b^ | 4.000 | 8.000 | .000 | .984 |

| Each F tests the multivariate effect of Extraction_method. These tests are based on the linearly independent pairwise comparisons among the estimated marginal means.^a^ |
| --- |
| a. Strain = V. cholerae |
| b. Exact statistic |

**Profile Plots**


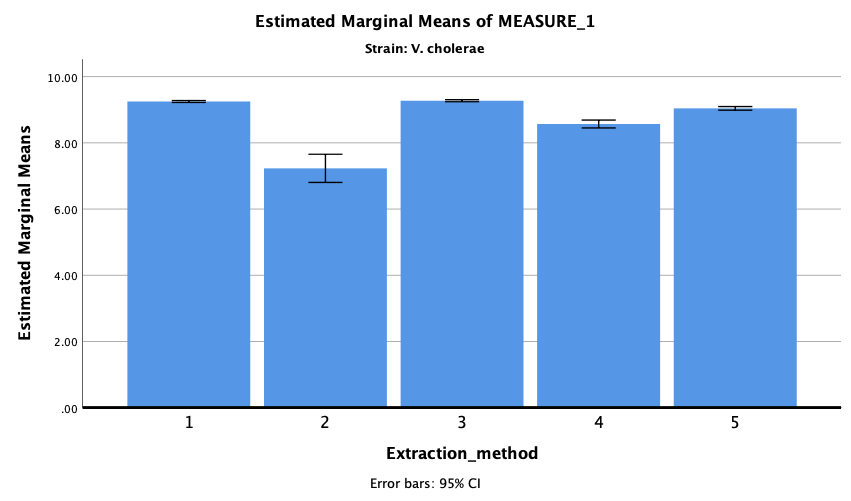

Supplement: Supplementary file 2 — Supplementary Materials on Statistical Analysis [file 41598_2019_50246_MOESM2_ESM.docx]
